# A correlative study of the genomic underpinning of virulence traits and drug tolerance of *Candida auris*

**DOI:** 10.1128/iai.00103-24

**Published:** 2024-05-09

**Authors:** Bo Yang, Benjamin Vaisvil, Daniel Schmitt, Joseph Collins, Eric Young, Vinayak Kapatral, Reeta Rao

**Affiliations:** 1 Department of Biology and Biotechnology, Worcester Polytechnic Institute, Worcester, Massachusetts, USA; 2 Igenbio, Inc., Chicago, Illinois, USA; 3 Department of Chemical Engineering, Worcester Polytechnic Institute, Worcester, Massachusetts, USA; Tulane University, New Orleans, Louisiana, USA

**Keywords:** *Candida auris*, genomic deletion, sugar assimilation, virulence factors, macrophage interaction, cell wall, drug tolerance

## Abstract

*Candida auris* is an opportunistic fungal pathogen with high mortality rates which presents a clear threat to public health. The risk of *C. auris* infection is high because it can colonize the body, resist antifungal treatment, and evade the immune system. The genetic mechanisms for these traits are not well known. Identifying them could lead to new targets for new treatments. To this end, we present an analysis of the genetics and gene expression patterns of *C. auris* carbon metabolism, drug resistance, and macrophage interaction. We chose to study two *C. auris* isolates simultaneously, one drug sensitive (B11220 from Clade II) and one drug resistant (B11221 from Clade III). Comparing the genomes, we confirm the previously reported finding that B11220 was missing a 12.8 kb region on chromosome VI. This region contains a gene cluster encoding proteins related to alternative sugar utilization. We show that B11221, which has the gene cluster, readily assimilates and utilizes D-galactose and L-rhamnose as compared to B11220, which harbors the deletion. B11221 exhibits increased adherence and drug resistance compared to B11220 when grown in these sugars. Transcriptomic analysis of both isolates grown on glucose or galactose showed that the gene cluster was upregulated when grown on D-galactose. These findings reinforce growing evidence of a link between metabolism and drug tolerance. B11221 resists phagocytosis by macrophages and exhibits decreased β-1,3-glucan exposure, a key determinant that allows Candida to evade the host immune system, as compared to B11220. In a transcriptomic analysis of both isolates co-cultured with macrophages, we find upregulation of genes associated with transport and transcription factors in B11221. Our studies show a positive correlation between membrane composition and immune evasion, alternate sugar utilization, and drug tolerance in *C. auris*.

## INTRODUCTION

*Candida auris* was first clinically isolated in 2009 and has been rapidly reported in the years since in over 30 countries worldwide (1, 2). Quickly, emerging as a public health threat, officially categorized as an urgent threat level by the Centers for Disease Control (3). Furthermore, the World Health Organization has noted that *C. auris* infection causes hospitalization and death and urges more research to enable drug discovery to treat this pathogen (4). The yeast can cause severe systemic infections, especially in immune-compromised individuals, with a mortality rate of greater than 40% due to limited treatment options (5–7). Effective treatment of infection of *C. auris* is challenging due to multi-drug resistance to antifungals (8), persistence in hospital environments with transmission within healthcare settings (8–10), and misidentifications that delay diagnosis (11, 12). From work on other *Candida* pathogens, it is known that virulence is a function of metabolic flexibility, host adherence, biofilm formation, filamentation, and secretion of hydrolytic enzymes (13). These virulence factors and fitness attributes of *C. auris* are not well understood. Here we attempt to connect genomic and transcriptomic results to key phenotypic virulence traits such as the ability to evade the host immune system, grow on non-canonical carbon sources, and drug tolerance.

*C. auris* has been phylogenetically placed within the *Clavispora*/*Candida* clade of the *Metschnikowiaceae* family of the order Saccharomycetales, yeasts that reproduce by budding (14). *C. auris* is in the CTG clade, a group of yeasts that translate the codon CTG to serine instead of leucine (15), although it is genetically distant from the other widely studied CTG clade species such as *C. albicans*, *C. tropicalis*, and *C. parapsilosis* (16). Previous studies suggested that *C. auris* emerged simultaneously and independently in different global regions (8). Based on genome sequence, geographic location of isolation, and drug resistance profile, *C. auris* isolates have been classified into five clades by geographic regions: Clade I (South Asian), Clade II (East Asian), Clade III (South African), Clade IV (South American) (8), and Clade V (Iran) (17). Genome sequence (16) of the first four clades reveals distinct separation by thousands of single-nucleotide polymorphisms (SNPs), although within each clade the isolates appear to be clonal (8). Clade V (Iran) is most recently discovered and is also separated from the other clades by >200,000 SNPs (17). Genome assemblies were completed first from Clade I isolates, such as Ci6684 (18), and five isolates from an outbreak in the United Kingdom (19). *C. auris* genomes have been mapped into seven chromosomes using optical maps, which were then confirmed by long-read sequencing and telomere-to-telomere assemblies (20). As of this publication, a single isolate from Clade V has been sequenced and its genome is highly syntenic with those of Clades I, III, and IV, which are the outbreak-causing clades (17, 20).

The CDC reports *C. auris* is multidrug resistant with 90% of the isolates being resistant to at least one antifungal agent and 30% being resistant to at least two antifungal drugs (21). Antifungals currently in clinical use fall into four major classes: azoles, polyenes, allylamines, and echinocandins (22). The first three classes inhibit ergosterol (23), an essential component of the fungal cell membrane, while echinocandins inhibit β-1,3-glucan (24), an essential component of the fungal cell wall. Both ergosterol and β-1,3-glucan are not present in mammals. Susceptibility testing indicates a disparity in drug effectiveness. One study has shown that over 90% of *C. auris* isolates are resistant to azoles, 10%–35% are resistant to the polyene amphotericin B, and 4%–7% are resistant to echinocandins (8, 25, 26). Another report stated that 44% of *C. auris* isolates are resistant to the azole fluconazole, 15% to amphotericin B, and 3% are resistant to the echinocandin caspofungin (27). A large body of literature has established a clear connection between azole and polyene resistance to mutations in the ergosterol pathway, specifically *ERG11*, *ERG2, ERG3,* and *ERG6* (28). The main mechanisms of *C. auris* to azole resistance are *ERG11* gene mutation, overregulation of *ERG11,* and efflux pump overregulation (29). *ERG11* is one of the most studied genes in *C. auris* resistance, which encodes the lanosterol-14α-demethylase, the target protein of azoles action (29). Overexpression of the *ERG*1, *ERG*2, *ERG*3, *ERG*5, *ERG*6, and *ERG*13 genes are observed in amphotericin B-resistant *C. auris* strains compared to susceptible ones (30, 31). After administration of amphotericin B to *C. auris*, one strain showed induced expression of five genes that are involved in the ergosterol biosynthesis pathway (*MVD*, *ERG2*, *ERG1*, *ERG6,* and *ERG13*) (16). Other mechanisms have been proposed, like variation in *ERG11* copy number, overexpression of efflux pumps, *HSP90* thermal shock protein, and changes in membrane composition that protect the target (29, 31). While in resistance to echinocandins, *C. auris* has an *FKS1* gene that encodes for β-1,3-d-glucan synthase that shares the two hot spots for resistance mutations in *Candida* spp. (32). Considering the mechanism of action of antifungals, alterations in the ergosterol pathway contribute to the primary incidence of multidrug resistance in *C. auris*. The fact that only a small number of strains are resistant to echinocandins highlights β-1,3-glucan biosynthesis as the most effective target pathway. As a result, echinocandins have become the drugs of choice for primary treatment of invasive *C. auris* infections (33). Further research on other molecular mechanisms of drug resistance in *C. auris* is needed to develop alternate therapies.

Antifungal drug tolerance has been proposed to lead to the evolution of drug resistance (34). Drug tolerance is defined as the ability of a subpopulation of drug-susceptible fungi to grow slowly when exposed to a drug above its minimum inhibitory concentration (MIC) (35). In the laboratory, tolerance is quantified using disk diffusion assays on solid media or broth microdilution assays after 48 hours of growth (35). In disk diffusion assays, drug-tolerant cells can be visualized as they grow inside the zone of inhibition. In a clinical setting, this manifests as patients with recurrent infections after treatment (36). Thus, distinguishing drug tolerance from resistance is important to provide options for treatment failure. The genetic underpinning of drug tolerance in yeasts is not well known, although point mutations, aneuploidy, loss of hetero-resistance, growth conditions, stress response pathways, and mutations in *TAC1*, a positive transcriptional regulator of efflux pumps, have all been implicated (37, 38).

The ability to survive on abiotic surfaces with limited nutrients and colonize glucose-limited niches is a key virulence factor for *C. auris*. Adaptation to different carbon sources has been connected to Candida virulence. Their ability to assimilate available nutrients allows them to respond to local environmental stressors and host defenses (39) giving them a competitive edge over other pathogens (40). Compared to glucose, *C. albicans* grown on lactate has a thinner cell wall with reduced β-1,3-glucan and chitin, enhancing antifungal resistance (41). Furthermore, they are better at evading the host immune system—cells are less likely to be taken up by macrophages and are more efficient at killing and escaping them (42). Acetate-grown *C. glabrata* are more susceptible to fluconazole, generate less robust biofilms, and are more susceptible to macrophage killing, possibly due to upregulation of carboxylate transporters and a potential role of *FPS1* and *ADY2a* in the phagocytosis process (43).

Host-pathogen interactions are mediated through the recognition of pathogen-associated molecular patterns (PAMPs) by pattern recognition receptors (PRRs) found in the immune cells. PAMPs derived from fungal pathogens are specifically recognized by PRRs, occurring at the interface between the immune cell membrane and the fungal cell wall. The major fungal PAMPs on the fungal cell surface include β-glucan, mannan, and chitin, which are critical components of the fungal cell wall (44). These have not been well characterized in *C. auris*. Polysaccharide fibrils of β-(1,3)-glucan covalently linked to β-(1,6)-glucan and chitin compose the core structure of *Candida* spp. cell wall and function as a scaffold for external proteins (45). β-glucan layers lie between chitin and mannan layers and trigger strong host inflammatory responses (46); however, it is normally masked by the less pro-inflammatory mannan layer but can be exposed during infection (47, 48). β-glucan recognition by its receptor, dectin-1, is required to control systemic infections (49) and plays a major role in anti-*Candida* immune responses (44). Its biosynthesis is critically dependent on β-(1,3)-glucan synthase activity [encoded by *FKS1* and *FKS2* genes (50)], which is the target of echinocandins such as caspofungin.

In this study, we used two *C. auris* isolates that have different antifungal resistance traits and are representatives of the most prevalent *C. auris* clades among reported cases (51). *C. auris* B11220 (Clade II) is susceptible to all antifungal drugs and is not associated with outbreaks, whereas *C. auris* B11221 (Clade III) is associated with multiple outbreaks, invasive, and multidrug-resistant infections. *C. auris* B11221, which retains the gene cluster missing in B11220^Δ^, is more drug resistant to caspofungin. The MIC-50 (MIC required to inhibit the growth of 50%) to caspofungin is 0.25 µg/mL for B11221 and only 0.125 µg/mL for B11220, according to the CDC.

A comparison of the two *C. auris* genomes revealed a gene cluster annotated as an L-rhamnose utilization cluster was missing in B11220 but found in B11221. B11221 uses alternate sugars more readily, exhibits greater drug tolerance, and survives encounters with host immune cells better than B11220. Our transcriptomic studies reveal that B11221 grown in D-galactose upregulates genes associated with the ribosomal complex, translation factors, protein metabolism, and carbohydrate metabolism. Several ORFs unique to B11221 were also found to function in stress response, cell wall biosynthesis, and carbohydrate metabolism. Transcriptomics studies of interaction between these isolates with macrophages reveal upregulation of transport-related transcription factors in B11221, which may contribute to its higher stress tolerance and survival in the host.

## MATERIALS AND METHODS

### Candida growth

*C. auris* isolates from frozen stock (−80°C) were streaked out on yeast-peptone-dextrose (YPD) agar and incubated at 30°C for 24 hours and subsequently stored at 4°C prior to liquid culture. A single colony of each isolate was picked and grown in 50 mL YPD medium at 30°C at 200  rpm for 16 hours. The cells were pelleted by centrifugation (3,000  ×  *g*) and washed three times in phosphate-buffered saline (PBS). The cells were then adjusted to the desired concentration after measurement with an optical spectrometer and resuspended in selected media for each assay, as described in each result section.

For growth assays, each well of a flat-bottom 96-well polystyrene plate was loaded with 200 µL of inoculum of 0.05 OD/mL in Synthetic Complete (SC) medium. Different carbon sources were added to a final concentration of 2%. Antifungal drugs were added as described. The experiments were conducted in triplicates incubated at 30°C for 45 hours on a shaker and measured at 600 nm every 15 min in a BioTek Synergy H1 plate reader. The growth curves were fitted using a non-linear model in Graphpad.

### Genome sequencing and analysis

#### Genome sequence and assembly

High-molecular-weight genomic DNA was isolated by a modified Promega’s Genomic DNA Isolation Kit (Promega, A1120) (52). Nanopore libraries were prepared with the Rapid Barcoding Kit (ONT, SQK-RBK004). Illumina libraries were prepared with the Nextera DNA Flex Library Prep Kit (Illumina, 20018704) along with the Nextera DNA CD Indexes (Illumina, 20018707). Nanopore sequencing was performed with the Oxford Nanopore MinION and MinKNOW software, and the resulting fastq files were demultiplexed using EPI2ME (Metrichor, Oxford, UK). Illumina sequencing was performed with Local Run Manager software on the iSeq 100 machine. A GENERATEFASTQ run was initiated and run with the parameters of Type: paired-end, read lengths: 151, and index reads: 2. The reads were demultiplexed using the native software on the iSeq 100 machine. The reads were independently assembled using the Prymetime (v0.2) pipeline (53), which is a hybrid assembler using long and short reads.

#### Sequence analysis

The genomes of two *C. auris* isolates were aligned to each other. Genome alignment was performed with ERGO’s Genome Align tool which uses BLASTN (54) with an expected value threshold of 1E-180 to identify blocks of homology, which are then visually inspected on a genome browser. Genes were predicted on the two assemblies using BRAKER (55) incorporating the RNA-Sequencing described in section Transcriptional Profiling by RNA-Sequencing, as well as fungal protein evidence derived from OrthoDB release 10 (56). Gene functions were assigned *via* protein similarity to orthologs with functions using the ERGO database as detailed in Overbeek et al. (57). In addition, protein domain analysis was performed using the NCBI Conserved Domain Database (58) as verified through manual curation. The predicted amino-acids bidirectional Best Hits (BBH) were computed to allow the mapping of names to the public B11221 reference where possible. Genes without a BBH have identifiers that start with “RTCAU.”

### Carbohydrate assay

The Total Carbohydrate Assay Kit (Sigma Catalog Number MAK104) was used for this assay. PBS-washed overnight cultures of *C. auris* isolates were inoculated to SC media, respectively, with 2% D-glucose, 2% D-galactose, and 2% L-rhamnose, at a concentration of 0.1 at OD_600_. Then the cultures were distributed to a 96-well plate at 40 µL per well. Media without *Candida* cells were used as controls. Standard curves were created using 2 mg/mL standard solutions. All conditions were in triplicate, incubated at 30°C for 24 hours. Reaction assays were followed as described by the protocol provided in the Kit. Colorimetric detection was taken at the end of assay at an absorbance of 490 nm to measure carbohydrates that remained in the media after cell growth. Carbohydrate consumption by growth was calculated by subtracting the remaining sugar from before growth.

### Surface adhesion assay

PBS-washed overnight cultures of *C. auris* isolates were inoculated to SC media, respectively, with 2% D-glucose, 2% D-galactose, and 2% L-rhamnose, at a concentration of 0.5 OD_600_. Cell suspension (200 µL) was aliquoted into each well of a flat-bottom 96-well polystyrene plate. Each condition was repeated in triplicate. The plates were covered and incubated at 37°C for 4 hours. After incubation, the unattached cells were discarded and 40 µL of 0.5% crystal violet (CV) solution was added to each well incubated for 45 minutes at room temperature to stain the attached *Candida* cells to the polystyrene plates. The excess dye was discarded, and the plates were washed six times with diH2O and gently tapped onto a paper towel to remove residual diH2O. To dissolve the CV in the attached cells, 200 µL of 75% methanol was added to each well and the plates were incubated at room temperature for 30 minutes. The absorbance of adhesion-retained CV dye was measured at 590 nm in Victor3 plate reader (PerkinElmer) and used as a measurement of adhesion to the plastic surface.

### Caspofungin susceptibility by disk diffusion assay

Overnight cultures of *C. auris* isolates were washed in PBS and were normalized to 0.5 OD_600_ in PBS. About 600 µL was placed on each SC agar containing 2% D-glucose, 2% D-galactose, and 2% L-rhamnose in petri dishes, respectively. L-shape spreader was immediately used to spread the culture evenly on agar. After drying plates for 1 hour, one paper disk saturated with caspofungin solution (4 mg/mL in DMSO, 5 µL) was placed in the center of each plate. These plates, in triplicate, were kept inverted at 30°C incubator. The zone of growth inhibition surrounding the filters (halo) was observed and photographed after 24 to 72 hours of incubation.

### Cell survival by “*ex vivo”* macrophage killing assay

Murine macrophage cell lines derived from the mouse line NR-9456 (BEI resources) were thawed and grown in Dulbecco’s Modified Eagle Medium (DMEM) plus 10% fetal bovine serum (FBS) to reach over 95% confluence. *Candida* cells were grown in YPD liquid media at 30°C overnight prior to the infection experiment, then acclimated to macrophage media (DMEM +10% FBS) at 37°C for 1 hour prior to the infection. On the day of the infection experiment, macrophages were seeded in 24-well plates at a density of 0.5 × 10^6^ cells/mL and incubated at 37°C for 4 hours for cells to attach to the plate bottom. The cells were counted by hemocytometer and seeded to each well in a ratio of 1 *Candida* cell to 15 macrophage cells. The mixture plates were then incubated at 37°C (5% CO_2_) for 4 and 8 hours. The cultures were then scraped down from the wells using cell scrapers. The contents from each well were transferred to the collection tubes with 0.02% Triton X-100 to osmotically lyse the macrophages. A serial dilution was quickly performed to make the suspension diluted to 10^−4^. 150 µL of the dilution was spread on YPD agar plates and incubated at 30°C for 24 hours to make colony forming units (CFU). *Candida* survival was calculated by taking the ratio of CFU obtained from *Candida* and macrophage mixture to that obtained from *Candida* alone condition. Each condition was repeated in triplicates.

### Quantification of β-1,3-glucan

#### Total cellular levels of β-1,3-glucan

*Candida* isolates were grown overnight and diluted to an OD_600_ to1. Then the diluted cultures were killed by incubation in a heat block at 100°C for 5 min. 100 µL of each isolate was added to 1.5 mL EP tubes. Tubes were centrifuged for 5 min at 3,000 × *g*. Harvested cells were resuspended in 50 µL PBS containing 2% BSA and incubated at room temperature for 1 hour using a swinging mixer. Tubes were centrifuged for 5 min at 3,000 × *g* and cells were resuspended in 50 µL of the antibody solution (antibody against β-1,3-glucan, Biosupplies, Cat. No. 4002), then incubated at 37°C for 2 hours on a swinging mixer. Tubes were centrifuged for 3 min at 3,000 × *g* and wells were washed three times with PBS containing 0.05% Tween-20 (PBS-T). Cells were then resuspended in 50 µL of the secondary antibody solution (1 µg/mL Abcam rabbit polyclonal anti-mouse 97046, 1:1,000). Tubes were incubated at 37°C for 1 hour on a swinging mixer/belly dancer. These tubes were centrifuged for 5 min at 3,000 × *g,* were washed three times with PBS-T, and finally resuspended in 50 µL/well chemiluminescent peroxidase substrate (Supersignal West Pico ThermoFisher Cat No. 34077). The samples were then transferred to a reading plate, and luminescence was measured in a Victor3 plate reader (PerkinElmer).

#### β-1, 3-glucan surface exposure

*Candida* isolates were grown overnight, washed, and diluted to an OD_600_ to 0.6. Each sample was blocked with 3% BSA in PBS then stained anti-β (1,3)-glucan antibody (Biosupplies Australia Pty Ltd., Australia) followed by the secondary antibody of anti-Mouse IgG (H + L) Alexa Fluor 488 (ThermoFisher). The level of β-1,3-glucan exposure on the cell surface was then quantified on a Beckman Coulter CytoFlex 3-laser cytometer. Controls included single-stained and unstained samples for each isolate. Flow cytometry data were analyzed using the Flowjo software to calculate the relative amounts of cell wall β-1,3-glucan.

### Transcriptional profiling by RNA-sequencing

#### Sample preparation

##### Carbon source study

Overnight cultures of *Candida* isolates were washed three times by PBS. An OD_600_ at 15 of each isolate was inoculated to SC media, respectively, containing 2% D-glucose and 2% D-galactose, in three replicates. All cultures were incubated at 30°C on a shaker at 220 rpm for 4 hours. The cultures were spun down to remove supernatant and pellets were immediately transferred to liquid nitrogen and stored at −80°C.

##### Host-pathogen interaction

The assay was followed similarly as described above for *Candida* survival. Modification was made to mix each *Candida* isolate and macrophage cells to each well in a ratio of 1:1 *Candida* cell to macrophage cells in DMEM containing 10% FBS. The mixture cultures in 24-well plates were incubated at 37°C (5% CO_2_) for 4 hours. The cultures were then scraped down from the wells using a cell scrapper. The contents from each well were transferred to collection tubes and then centrifuged to remove the supernatant. Cell pellets were immediately transferred to liquid nitrogen and stored in the −80°C freezer. In total, there were three replicates of each Candida isolate exposed to macrophage cells and two replicates of the Candida isolates without the presence of macrophages for a total of 10 samples.

### RNA sequencing

Total RNA was extracted using the Qiagen RNeasy kit following the manufacturer’s directions; furthermore, polyA mRNA was used as input to SMARTer Stranded RNA-Seq Kit (Takara Biosystems) according to the manufacturer’s instructions for cDNA library preparation. The library was sequenced on Illumina HiSeq 2 × 150 obtaining approximately 593 million reads. The sequenced reads were checked for quality using ERGO’s (57) read QC workflow. Reads were then quantified using kallisto (59) through ERGO’s RNA-Seq workflow. The transcript abundances were summarized by the tx2gene R package (60) and subsequently converted into counts. Gene counts were imported into DESeq2 R package (61) and tested for differential expression using DESeq2’s deseq function. Genes were considered significantly differentially with a log2 fold change greater than 1 and a false discovery rate (FDR)-adjusted *P* value less than 0.05. Genes identified as differentially expressed in all carbon sources were filtered. Pathway enrichment analysis was performed using GAGE (62) in the ERGO suite.

## RESULTS

### A 12.8 kb deletion region encodes genes involved in alternate sugar utilization

**Fig 1:**
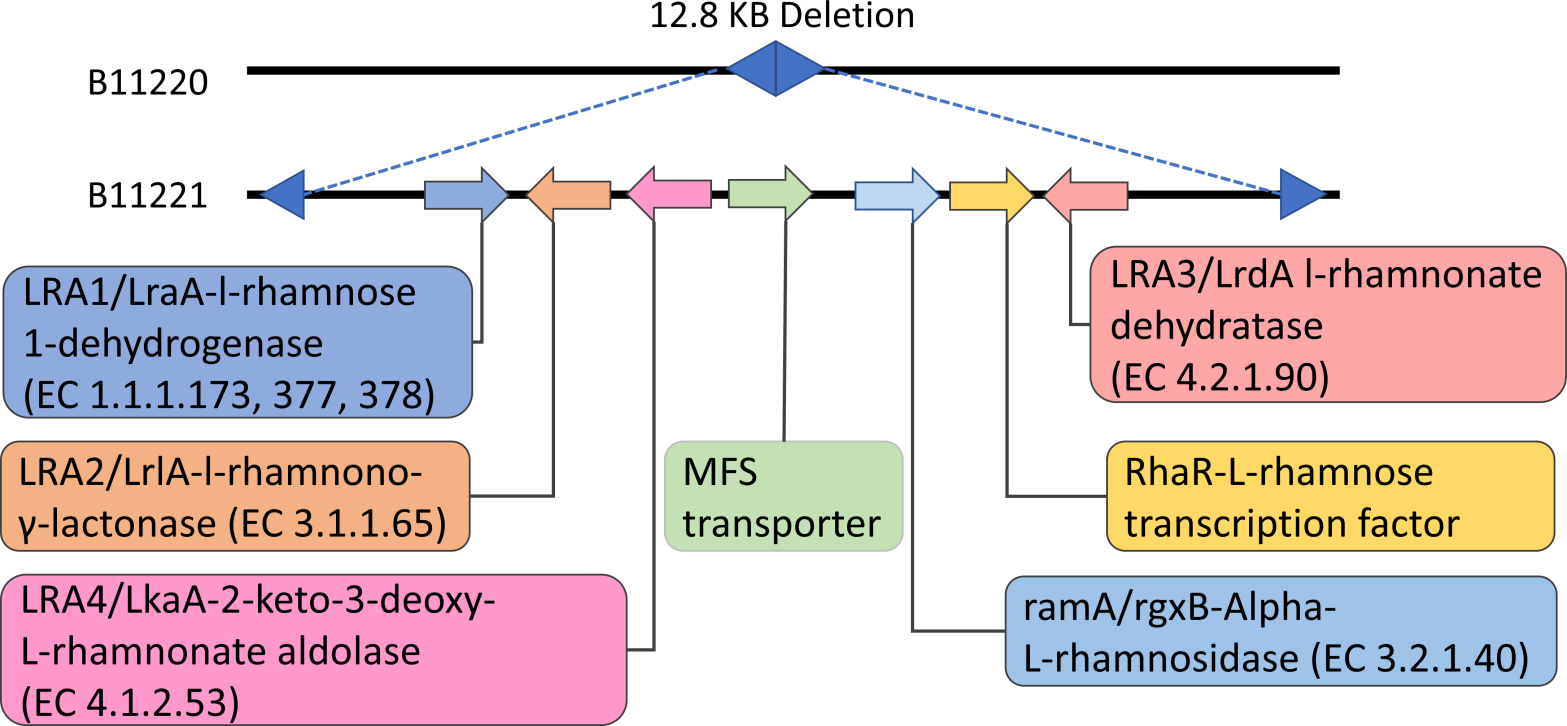
12.8 kb region absent from B11220^∆^ compared to B11221. Location and regions of the seven closest ORFs for L-rhamnose digestion. Black lines are homologous between the two genomes.

We compared the genome sequences of *C. auris* isolates B11220 and B11221, both have previously been sequenced (20). B11220 is from Clade II (East Asia) and sensitive to the three major classes of antifungal drugs, whereas isolate B11221, from Clade III (South Africa), is resistant to all three major classes of antifungal drugs. We re-sequenced these isolates using the Prymetime (v0.2) pipeline which uses both long reads and short reads to obtain a more contiguous genome assembly (53). Using ERGO’s genome align tool, we confirmed a previously identified ~12.8 kb region is present in B11221 and absent in B11220 (20). We confirmed, *via* BLAST searches of the other available isolate genome sequences, that it is absent in Clades I, II, and IV isolates and present in Clades III and V (20). This region contains seven predicted open reading frames (ORFs) that potentially code for the transport and degradation of alternate sugars in general and have homologs predicted to metabolize L-rhamnose (Fig. 1) in *Aspergillus spp* (63–65). These ORFs are identified as (CJI97_002171) *LRA1* (LraA-L-rhamnose 1-dehydrogenase, EC 1.1.1.173), (CJI97_002177) *LRA2* (LrlA-l-rhamnono-γ-lactonase, EC 3.1.1.65), *LRA4* (LkaA-2-keto-3-deoxy-L-rhamnonate aldolase, EC 4.1.2.53), (CJI97_002174) major facilitator superfamily (MFS) sugar transporter, (CJI97_002175) *ramA* (rgxB-Alpha-L-rhamnosidase, EC 3.2.1.40), (CJI97_002176) *RhaR* [transcriptional regulator involved in the release and catabolism of the methyl-pentose (66)], and (CJI97_002177) *LRA3* (LrdA l-rhamnonate dehydratase, EC 4.2.1.90). For reader convenience, we will identify the isolate with the deletion with a delta superscript (B11220^Δ^) to distinguish it from B11221 which contains the gene cluster. We hypothesize that this genomic difference could be associated with phenotypic variation of *C. auris*. In this manuscript, we demonstrate a possible connection between these seven genes regarding carbon utilization and drug tolerance.

L-rhamnose (l-6-deoxy-mannose) is a C6 sugar that can be used as a carbon and energy source in several microorganisms (67). There are two pathways known for L-Rha catabolism: one with phosphorylated intermediates described in bacteria, and the other one without phosphorylated intermediates described in yeast species like *Pullularia pullulans* (68), *Pichia stipitis*, and *Debaryomyces polymorphus* (69). The enzymes in the fungal L-Rha catabolism pathway include l-rhamnose-1-dehydrogenase (EC 1.1.1.173), l-rhamnono-1,4-lactonase (EC 3.1.1.65), l-rhamnonate dehydratase (EC 4.2.1.90), and l-erythro-3,6-dideoxyhexulosonate aldolase (EC 4.1.2.-) (67). These genes are involved in L-Rha transport from extracellular media and convert in four enzymatic steps to pyruvate and feed into the TCA cycle (70).

**Fig 2:**
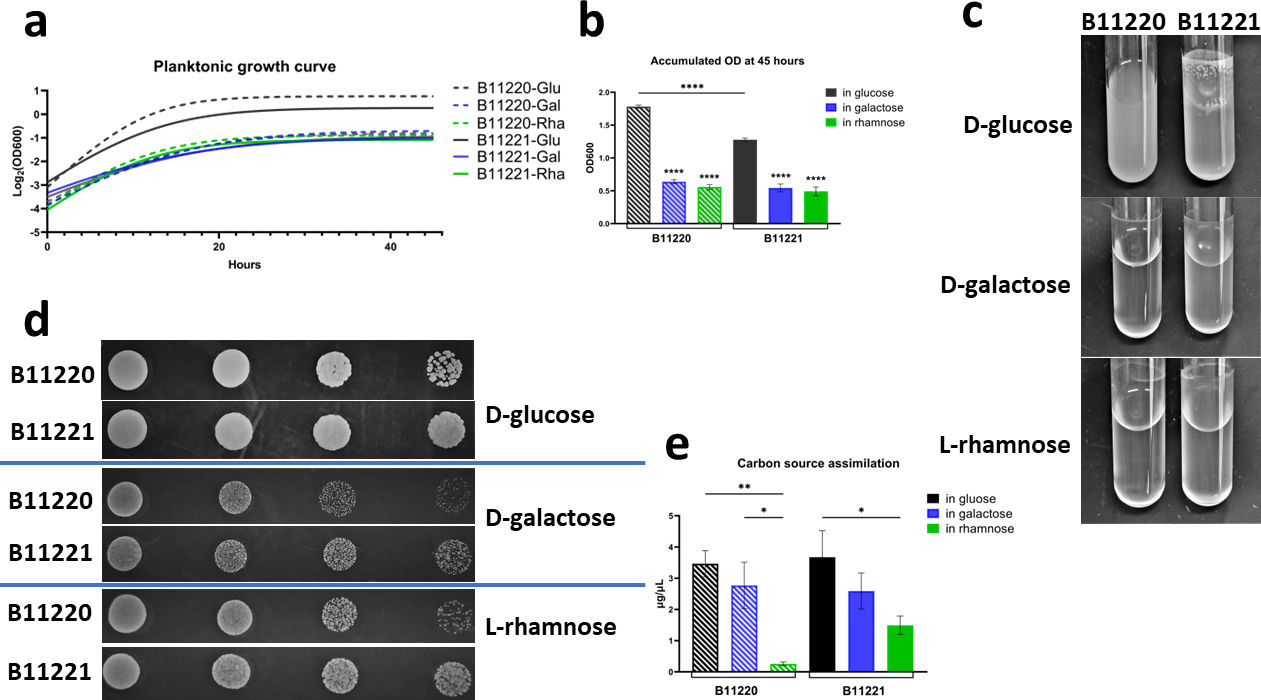
Growth of two *C. auris* isolates in three sugars. (**a**) Planktonic growth of B11220^Δ^ (blue) and B11221 (green) in liquid SC media. Curve fit with a non-linear model. Logged. (**b**) Endpoint OD_600_ comparison of growth in liquid culture tubes at 45 hours. Underlined asterisk marks indicate significant differences of the same sugar type between two isolates, and asterisk marks without underline indicate significant differences between different sugars within one isolate (****P < 0.0001). (**c**) Liquid culture overnight grown in SC media with glucose, galactose, and rhamnose in glass tubes. (**d**) Serial diluted colonies growth of B11220^Δ^ (top rows) and B11221 (bottom rows) on SC agar in three sugars. OD_600_ of colonies from left to right are 0.1, 0.01,0.001, and 0.0001. (**e**) Assimilation of three sugars as carbon sources by two *C. auris* isolates using Total Carbohydrate Assay Kit from Sigma. Tukey’s multiple comparisons test (****P < 0.0001, **P < 0.005, *P < 0.05).

We hypothesized that this gene cluster is involved in the metabolism of non-canonical sugar sources since many proteins involved in the transport and catabolism of alternative sugars are promiscuous (71). To find whether the ORFs affect alternative sugar metabolism for *C. auris*, we performed spot assays for D-glucose (control), L-arabinose, D-galactose (27), α-lactose, D-maltose, L-rhamnose, sucrose, and D-xylose (Fig. S1). There was no difference observed between the two isolates under those conditions. However, we further characterized growth on L-rhamnose and D-galactose by multiple quantitative methods including measuring liquid culture growth curves, measuring the growth on solid agar media, and carbon source assimilation analysis (Fig. 2). Our results show that there was no significant difference in the growth curves between B11220^Δ^ and B11221 in D-galactose or L-rhamnose in liquid cultures (Fig. 2a). When grown in the presence of D-glucose, B11220^Δ^ accumulated to a higher OD_600_ than B11221 in liquid media (Fig. 2b). Overnight liquid culture of B11221 was clumpy in D-glucose but the aggregative phenotype was not observed in D-galactose or L-rhamnose, while B11220^Δ^ cultures grew planktonically without aggregates in three sugars conditions (Fig. 2c). Differences of candida growth phenotypes usually can be more obviously observed when growing in solid media than in liquid form. When grown on agar plates in solid cultures, small colonies as well as a decreased number of cell colonies were observed in both B11220^Δ^ and B11221 when in D-galactose and L-rhamnose as the carbon source as compared to D-glucose (Fig. 2d). Isolate B11220^Δ^ formed fewer CFU as compared to B11221 in each sugar at OD_600_ dilution of 0.001 and 0.0001 (Fig. 2d). Assimilation of D-glucose as well as D-galactose was indistinguishable between the two isolates, but L-rhamnose assimilation was significantly different (Fig. 2e). These results show a positive correlation between the missing genes with L-rhamnose assimilation in *C. auris*, suggesting an alternate pathway present that allows B11220^Δ^ to grow on L-rhamnose. Thus, we hypothesize that the presence of this cluster enhances, but is not necessary for L-rhamnose utilization.

### Alternative carbon sources increase drug tolerance of *C. auris* isolate B11221

Alternative carbon sources can modulate the sensitivity of *Candida* species to antifungal drugs (39). Therefore, we measured the drug sensitivity of *C. auris* B11220^Δ^ and B11221 when grown in D-glucose, D-galactose, and L-rhamnose. We exposed the microbes to a representative of each of the three classes of antifungal agents, fluconazole (azole), amphotericin B (polyene), and caspofungin (echinocandin). We tested the MICs of the two isolates with the E-test assay and determined the MIC values (in μg/mL) of fluconazole to be 8 for B11220^Δ^ versus 256 for B11221; of amphotericin B to be 0.25 for B11220^Δ^ versus 0.38 for B11221; of caspofungin to be 0.012 for B11220^Δ^ versus 0.38 for B11221. Based on these values and the information from CDC reports (72), we used fluconazole at 10 µg/mL for B11220^Δ^ and at 130 µg/mL for B11221; amphotericin B at 0.38 µg/mL for both isolates; and caspofungin at 0.2 µg/mL for B11220^Δ^ and 16 µg/mL for B11221.

**Fig 3:**
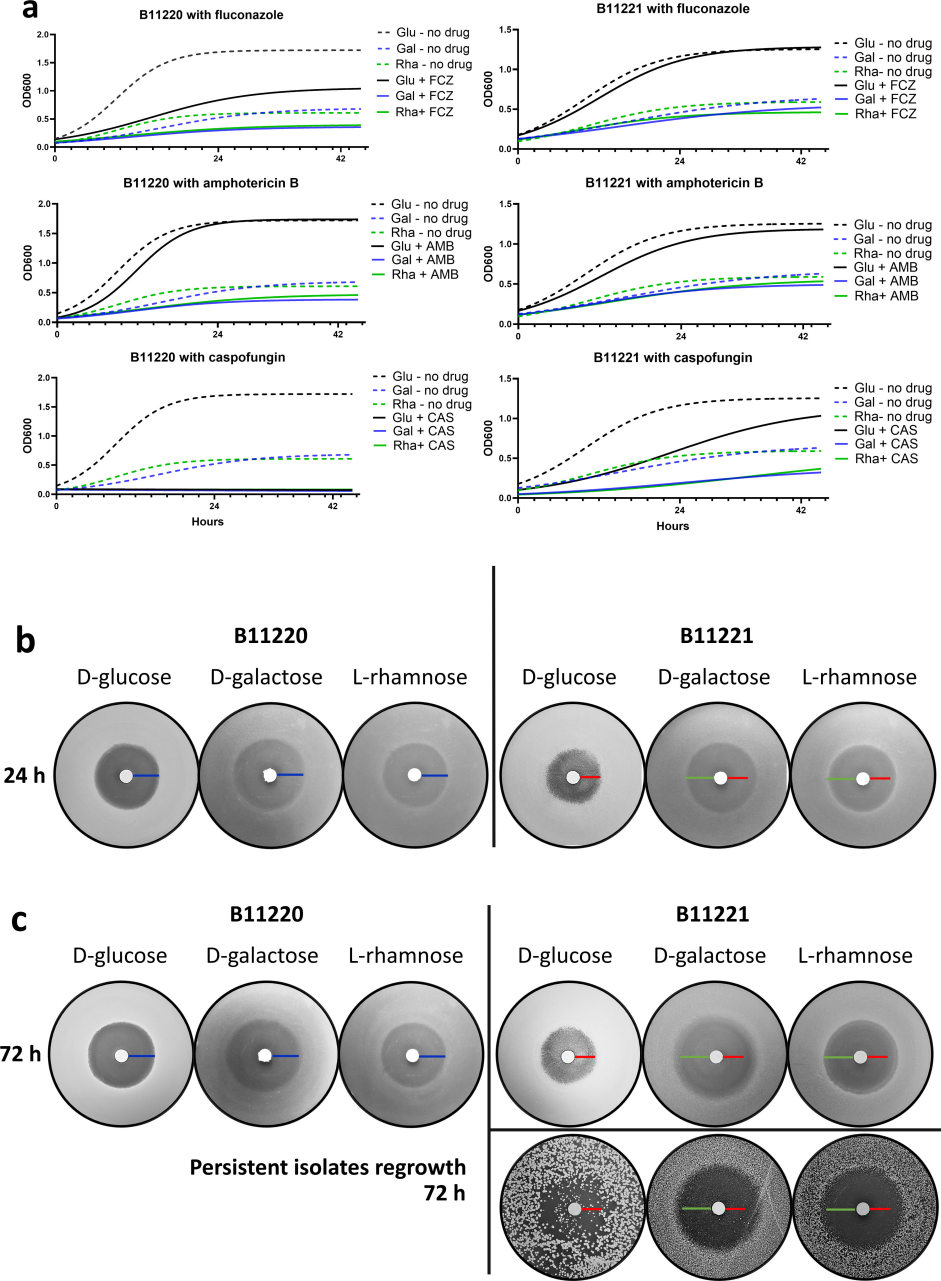
*C. auris* isolates growth in the presence of three antifungal drugs in three carbon sources. (**a**) Planktonic growth of two isolates in liquid SC media. Curve fitting with a non-linear model. Left panels: curves of B11220Δ. Right panels: curves of B11221. Top: in fluconazole, drug concentration was 10 µg/mL for B11220Δ, 130 μg/mL for B11221. Middle: in amphotericin B, a drug concentration was 0.38 μg/mL for both isolates. Bottom: in caspofungin, a drug concentration was 0.2 μg/mL for B11220Δ and 16 µg/mL for B11221. The red line indicates the duration at 24 hours. (**b**) Photographs from disk diffusion assay showing 24-hour growth of two *C. auris* isolates on SC agar in three carbon sources in the presence of caspofungin disks at concentrations of 1M. Left panel: B11220^Δ^, right panel: B11221. Agar plates were incubated at 30°C. Scale bars in blue measure 9.14 mm (0.36″), red measure 7.11 mm (0.28″), and green measure 10.16 mm (0.4″). (**c**) Disk diffusion assay showing 72-hour growth of two *C. auris* isolates on SC agar in three carbon sources in the presence of caspofungin disks at concentrations of 1M. Colonies of B11221 from the zone of inhibition were picked after 72 hours and grown again for 72 hours (right-bottom). Photos are representative of biological repeats of the experiment four times.

As expected, the growth of *C. auris* B11220^Δ^ and B11221 is affected by antifungals with every carbon source (Fig. 3a). Both isolates are equally inhibited by amphotericin B; however, B11220^Δ^ is particularly inhibited in fluconazole and does not grow in the presence of caspofungin, while *C. auris* B11221 is most inhibited by caspofungin but continues to grow (Fig. 3a). This is consistent with the finding that isolate B11221 is sensitive but tolerant to caspofungin (73).

To further investigate drug tolerance, we performed a disk diffusion assay for each isolate grown on all three carbon sources. Tolerance is shown by colonies within the zone of growth inhibition (or halo), which indicates slow growth at concentrations above the MIC, and is measured as a fraction of growth (FoG) (35, 36). In this assay, the clear zones formed by *C. auris* isolate B11220^Δ^ in each of the three sugars were comparable in size and maintained the area from 24 to 72 hours during growth, as indicated by the blue scale bar (Fig. 3b and c, left panels). However, isolate B11221 formed halos in D-galactose and L-rhamnose that were larger compared to that in D-glucose after 24 hours of growth (Fig. 3b and c, right panels), suggesting a lower MIC to caspofungin in these alternative sugars (35). This phenotype is consistent with the growth patterns we observed in aqueous media (Fig. 3a, bottom panel). After 48 hours, colonies appeared inside the zone of inhibition for *C. auris* isolate B11221 in all three sugars (Fig. 3c), indicating growth above the MIC and therefore drug tolerance (35). The drug tolerance phenotype was especially pronounced in D-galactose (Fig. 3c, right panel).

Drug-tolerant isolate B11221 colonies were then harvested and regrown on SC agar. Specifically, colonies from the zone of inhibition were taken after 72 hours and grown again for 72 hours in the presence of each of the three sugars with or without caspofungin (Fig. 3c, right-bottom panel). Compared with the first round of growth, D-glucose regrown colonies were bigger in size, maintaining a zone of inhibition, and tolerant isolates appeared as early as 24 hours of incubation. On D-galactose and L-rhamnose plates, the second-round inhibition zones were similar in size to the first-round suggesting their MIC to caspofungin remained. In addition to the zone area, there was no colony regrown when in L-rhamnose suggesting a loss of drug tolerance. Fewer tolerant colonies were able to develop within the inhibition zone in D-galactose, and these appeared to maintain a drug tolerance to caspofungin in the presence of D-galactose. Together, our results suggest *C. auris* B11221 is tolerant to caspofungin, especially in the presence of alternate sugars.

### Alternate carbon sources reduce adhesion and aggregation of *C. auris* B11221

**Fig 4:**
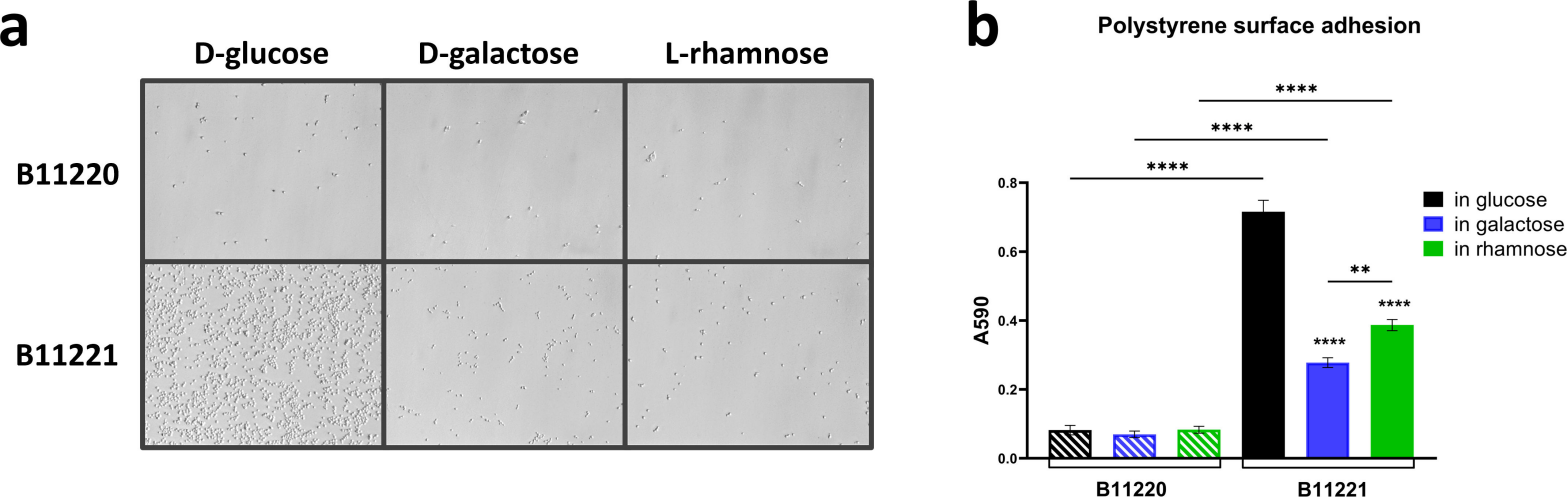
Surface adhesion characterization of B11220^Δ^ and B11221 in three carbon sources. (**a**) Microscopy of two *C. auris* isolates cells adhered to polystyrene plate bottom after washing off the liquid culture with three sugar sources. 400× magnification. (**b**) Quantification of two *C. auris* isolates cells adhered to the polystyrene plate bottom after 4 hours of liquid culture with three sugar sources. Error bars represent the standard error (SEM). Tukey’s multiple comparisons test (****P < 0.0001, **P < 0.005).

Adhesion and aggregation affect virulence *via* colonization, biofilm formation on surfaces, and ultimately dissemination to initiate infections at distal locations (74–78). Studies have shown that *C. auris* can survive on both moist and dry surfaces for long periods and still be cultured (79, 80), and it is more inclined to adhere and form biofilms on catheters as compared to *C. albicans* (81). The persistence of *C. auris* on abiotic surfaces presents opportunities for it to colonize and spread rapidly within healthcare facilities. We characterized the adhesion of B11220^Δ^ and B11221 to polystyrene and agar when grown on D-glucose, D-galactose, and L-rhamnose. The alternate sugars did not affect adhesion for B11220^Δ^ (Fig. 4), but B11221 adhered significantly less in D-galactose and L-rhamnose as compared to D-glucose (Fig. 4b). Furthermore, B11221 adhered better than B11220^Δ^ in all three carbon sources on polystyrene surface (Fig. 4a and b), as well as on agars (Fig. S2). In addition to adhesion, the aggregation phenotype of B11221 is also reduced in the presence of non-canonical sugars (Fig. 2c), which can be potentially caused by many factors including environmental stressors, host response, surface structure change, and transcriptional changes in genes involved in cell surface adhesion (82). Upon inspection of the genomes, we found an ORF (CJI97_002126), encoding cell wall agglutinin, was unique to isolate B11221 (Table 1) and not identified in isolate B11220^Δ^. This gene could potentially contribute to the aggregation and adhesion phenotypes associated with isolate B11221. Taken together, these data suggest a direct correlation between the utilization of alternative sugars and virulence phenotypes of aggregation and adhesion.

**TABLE 1:** ORFs unique to *C. auris* B11221 and their upregulation in two sugar sources after 4 hours as compared to PBS^*a*^

ORFs	Function	In D-glucose vs PBS		In D-galactose vs PBS		D-glucose vs D-galactose	
		log2 FC	q-value	log2 FC	q-value	Log2 FC	q-value
CJI97_002132	Formamidase	−2.84	6.16e-51	−1.92	1.01e-23	−0.917	8.55e-6
CJI97_002126	Cell wall agglutinin	3.28	4.45e-18	ns		2.39	8.51e-12
RTCAU38886 RTCAU37873	Hyphal-regulated cell wall protein/Exo-alpha sialidase	ns		4.26 7.03	4.47e-17 1.44e-9	−3.19 −6.21	1.03e-5 2.10e-13
CJI97_004171	Multidrug resistance ABC transporter ATP-binding/permease protein	ns		ns		ns	
CJI97_001781 CJI97_001080 RTCAU38895	GAL4 transcription factor	ns		ns		ns −0.880 ns	ns 1.97e-4 ns

^*a*^ FC: log2 fold change; q-value: FDR-adjusted *P*-value; ns: non-significant.

### Differentially expressed genes in *C. auris* B11220^Δ^ and B11221 with D-galactose

**Fig 5:**
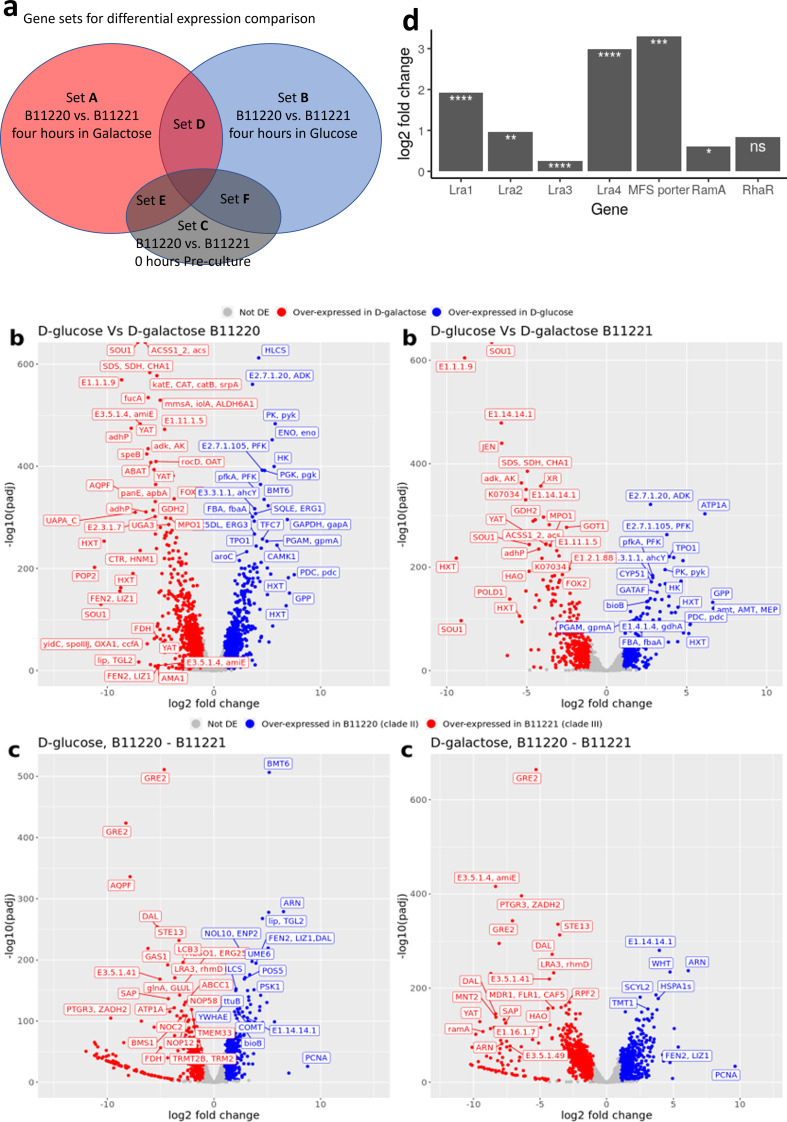
Transcriptome analysis of two *C. auris* isolates in carbon source of D-glucose and D-galactose. (**a**) Gene sets were chosen for differential expression comparison. Genes from Set A and Set B were compared for volcano plots, Set ABC was used for GO analysis, and Set DEF was excluded. (**b**) RNA-seq volcano plots of log2 fold changes in B11220^Δ^ (left) and in B11221 (right) in the presence of D-glucose (Glu) compared with D-galactose (Gal). Genes were differentially expressed (color coded) with a fold change > 1 and an FDR-adjusted *P* value < 0.05. Genes with a positive log2 fold change are over-expressed in D-glucose and under-expressed in D-galactose. Genes that have a negative log2 fold change are over-expressed in D-galactose and under-expressed in D-glucose. (**c**) RNA-seq volcano plots of log2 fold changes in B11220^Δ^ compared with B11221, respectively, in D-glucose (Glu, left) and in D-galactose (Gal, right). Genes were differentially expressed (color coded) with a fold change > 1 and an FDR-adjusted *P* value < 0.05. Genes with a positive log2 fold change are over-expressed in B11220^Δ^ and under-expressed in B11221. Genes that have a negative log2 fold change are over-expressed in B11221 and under-expressed in B11220^Δ^. *P*adj = adjusted *P*-value obtained from DESeq2. (**d**) Expression of seven L-rha ORFs in B11221 in the presence of galactose. The Y-axis represents Log2fold change. Asterisks denote False Discovery Rate adjusted *P*-values (q-value). **** q ≤1E−9, *** q ≤ 1E−4, ** q ≤ 1E−3, * q ≤ 0.05, ns not significant.

The reduced adhesion and increased tolerance to caspofungin of *C. auris* isolate B11221 as compared to B11220^Δ^ when grown in D-galactose suggests a metabolic shift in isolate B11221. First, we analyzed the differential gene expression of isolate B11220^Δ^ grown for 4 hours in 2% D-glucose versus 2% D-galactose. We found that the number of significantly (FDR-adjusted *P* < 0.05) differentially expressed genes (DEGs) was greater when in the presence of D-glucose than D-galactose. In all, 952 genes were over-expressed in the presence of D-glucose compared to 856 in the presence of D-galactose (Fig. 5b left; Fig. S3A). Genes that had the greatest over-expression in the presence of D-glucose compared to D-galactose include CJI97_001552 pyruvate decarboxylase EC 4.1.1.1 (7.53 FC, FDR-adjusted *P* 2.57e-82), CJI97_004441 DL-glycerol-3-phosphatase EC 3.1.3.21 (FC 7.08, FDR-adjusted *P* 1.17e-66), and two Hexose Transporters (CJI97_002024, CJI97_002023 FC 6.98 and 6.77; FDR-adjusted *P* 8.79e-80, 6.63e-56). Genes that had the greatest under-expression were CJI97_001009 aminotransferase (FC −11.2, FDR-adjusted *P* 1.31e-88), CJI97_003341 L-xylulose reductase (FC −10.6 FDR-adjusted *P* 3.89e-57), and CJI97_001794 low-affinity glucose/mannose transporter (FC −10.3, FDR-adjusted *P* 7.21e-111).

Next, we analyzed the differential gene expression of isolate B11221 grown under the same conditions as B11220^Δ^: 4 hours in D-galactose versus D-glucose. We found that the significantly (FDR-adjusted *P* < 0.05) DEGs were greater in the presence of D-galactose (500) versus D-glucose (328) (Fig. 5b right; Fig. S3A). The top over-expressed genes included CJI97_001552 pyruvate decarboxylase EC (4.1.1.1) (FC 5.23 FDR-adjusted *P* 8.31e-40), CJI97_000566 low-affinity zinc transporter (FC 6.8 FDR-adjusted *P* 1.51e-42), and CJI97_002683 ammonium transporter (FC 6.67 FDR-adjusted *P* 4.63e-53). The top under-expressed genes included CJI97_001794 low-affinity glucose transporter (FC −9.40 FDR-adjusted *P* 3.75e-95), CJI97_003341 L-xylulose reductase (FC −9.10 FDR-adjusted *P* 1.04e-42), and CJI97_003342 D-xylulose reductase EC 1.1.1.9 (FC −8.88 FDR-adjusted *P* 2.57e-263).

We further compared the four sets of DEGs: B11220^Δ^ upregulated in the presence of D-glucose compared to D-galactose, B11220^Δ^ upregulated in the presence of D-galactose compared to D-glucose, B11221 upregulated in the presence of D-glucose compared to D-galactose, and B11221 upregulated in the presence of D-galactose compared to D-glucose (Fig. 5b). We found that 493 genes were uniquely regulated in B11220^Δ^ in the presence of D-galactose compared to 139 in the presence of D-galactose in B11221. In all, 705 genes were uniquely regulated in B11220^Δ^ while in the presence of D-glucose compared to 79 unique to B11221, while 242 were shared between both strains (Fig. S3A).

Expression analysis between the isolates was examined when grown in different carbon sources. In D-glucose, 600 genes were significantly over-expressed in B11220^Δ^ (FDR-adjusted *P* < 0.05) compared to B11221, and 560 were under-expressed (Fig. 5c left; Fig. S3B). The greatest over-expressed genes were RTCAU15128 proliferating cell nuclear antigen (FC 8.78 FDR-adjusted *P* 5.08e-12), RTCAU00185 zinc peptidase family protein (FC 7.02 FDR-adjusted *P* 2.93e-7), and CJI97_004977 MFS transporter (FC 6.52 FDR-adjusted *P* 5.08e-12).

In cells grown in D-galactose, we found 665 genes significantly over-expressed in isolate B11220^Δ^ when compared to isolate B11221 with 775 being significantly under-expressed (Fig. 5c right; Fig. S3B). This analysis identified the top over-expressed genes as RTCAU15128 proliferating cell nuclear antigen (FC 9.64, FDR-adjusted *P* 1.79e-15) and MFS Transporters (FC 6.12, 5.37 FDR-adjusted *P* 1.19e-103, 2.93e-33). Those genes with the greatest under-expression included CJI97_004547 transcriptional regulator (FC −10.2, FDR-adjusted *P* 3.06e-17), CJI97_004170 hypothetical protein (FC −10.1, FDR-adjusted *P* 1.96e-16), and RTCAU37871 and RTCAU37885 hyphally regulated cell wall protein (FC -10.1,–9.82, FDR-adjusted *P* 4.95e-33, 5.65e-45).

Furthermore, we examined the ERGO gene ontology categories of the differentially expressed genes. The genes upregulated in B11221 were enriched in pathways associated with translation factors (Fig. S4B), protein metabolism (Eukaryotic Protein fate and biosynthesis), translation initiation factor activity, snoRNA binding, and the ribosomal complex (Fig. S4A and B). The genes upregulated in isolate B11220^Δ^ were enriched in genes associated with transport (Fig. S4B). The results suggest that isolate B11221 increases translation and protein production during D-galactose metabolism.

All seven genes of the putative L-rhamnose gene cluster were significantly upregulated in isolate B11221 when grown in D-galactose compared to D-glucose (Fig. 5d). The genes encoding an MFS transporter and *LRA4* (LkaA-2-keto-3-deoxy-L-rhamnonate aldolase, EC 4.1.2.53) increased expression about three log_2_ fold. *LRA1* (LraA-L-rhamnose 1-dehydrogenase, EC 1.1.1.173, 377, 378) increased two log_2_ fold. ORFs of *LRA2* (LrlA-l-rhamnono-γ-lactonase, EC 3.1.1.65), *LRA3* (LrdA L-rhamnonatedehydratase, EC 4.2.1.90) and *RamA* (rgxB-α-L-rhamnosidase, EC 3.2.1.40), and *RhaR* were also upregulated significantly in D-galactose. The upregulation of L-rhamnose degradation genes in the presence of D-galactose implies this gene cluster may be functionally involved in D-galactose assimilation in *C. auris* B11221. This finding suggests that these genes are promiscuous and may be involved in alternate sugar metabolism rather than reserved exclusively for L-rhamnose.

The expression of ORFs unique to B11221 was also studied by RNA sequencing in the presence of D-glucose and D-galactose (Table 1). CJI97_002126 cell wall agglutinin was unique to B11221. It was upregulated in D-glucose (3.28 log_2_ fold-change with q-value of 4.45e-18) versus PBS but not in D-galactose versus PBS after 4 hours of growth. Agglutinin-like sequence proteins (Als) are cell surface glycoproteins of *Candida* species that play a critical role in aggregation and mediate adherence and biofilm formation *in vitro* (83). Isolate B11221 showed aggregative phenotype when grown in a liquid medium in the presence of D-glucose but not D-galactose (Fig. 2c). A positive correlation between the upregulation of cell wall agglutinin in isolate B11221 in D-galactose may contribute to the difference in adhesion between the carbon sources for this isolate. Our future efforts will focus on testing this hypothesis when a knockout mutant is available.

An ORF (CJI97_002132) for formamidase (EC 3.5.1.49) was identified in isolate B11221 but not in isolate B11220^Δ^. The formamidase gene has been identified in other human pathogens such as *Paracoccidioides brasiliensis* and *Helicobacter pylori* whose function in pathogenesis is unknown but it is known to hydrolysis of formamide to produce formate and toxic ammonia gas. Formate can further be converted to oxalate to feed into the TCA cycle and is found on the surface of hyphal cells. Studies have confirmed that sera of patients with proven paracoccidioidomycosis recognize the protein (84). This gene was under-expressed in both D-glucose media compared to PBS (−2.84 log2 fold-change with FDR-adjusted *P*-value of 6.16e-51) and D-galactose media compared to PBS (−1.92 log2 fold-change with FDR-adjusted *P*-value of 1.01e-23).

Several ORFs unique to B11221 and located outside the 12.8 kb cluster were identified and functionally categorized (information is provided in supplement file tcau.expression.genelist.gos.csv). We found three ORFs (CJI97_001781, RTCAU38895, and CJI97_001080) in the *GAL4* transcription factor family, two ORFs (RTCAU38886 and RTCAU37873) for hyphal-regulated cell wall protein/exo-alpha sialidase (EC 3.2.1.18), and an ORF (CJI97_004171) for multidrug resistance ABC transporter ATP-binding/permease protein. These results, together with the biosynthetic gene cluster, point to increased galactose gene regulation in *C. auris* B11221, and the upregulated genes encode carbon source utilization, cell wall remodeling, and virulence genes. Therefore, alternative sugar metabolism and virulence traits may be interrelated in *C. auris*.

### B11221 survives encounters with macrophages better than B11220^Δ^ by upregulating transporters and transcription factors

**Fig 6:**
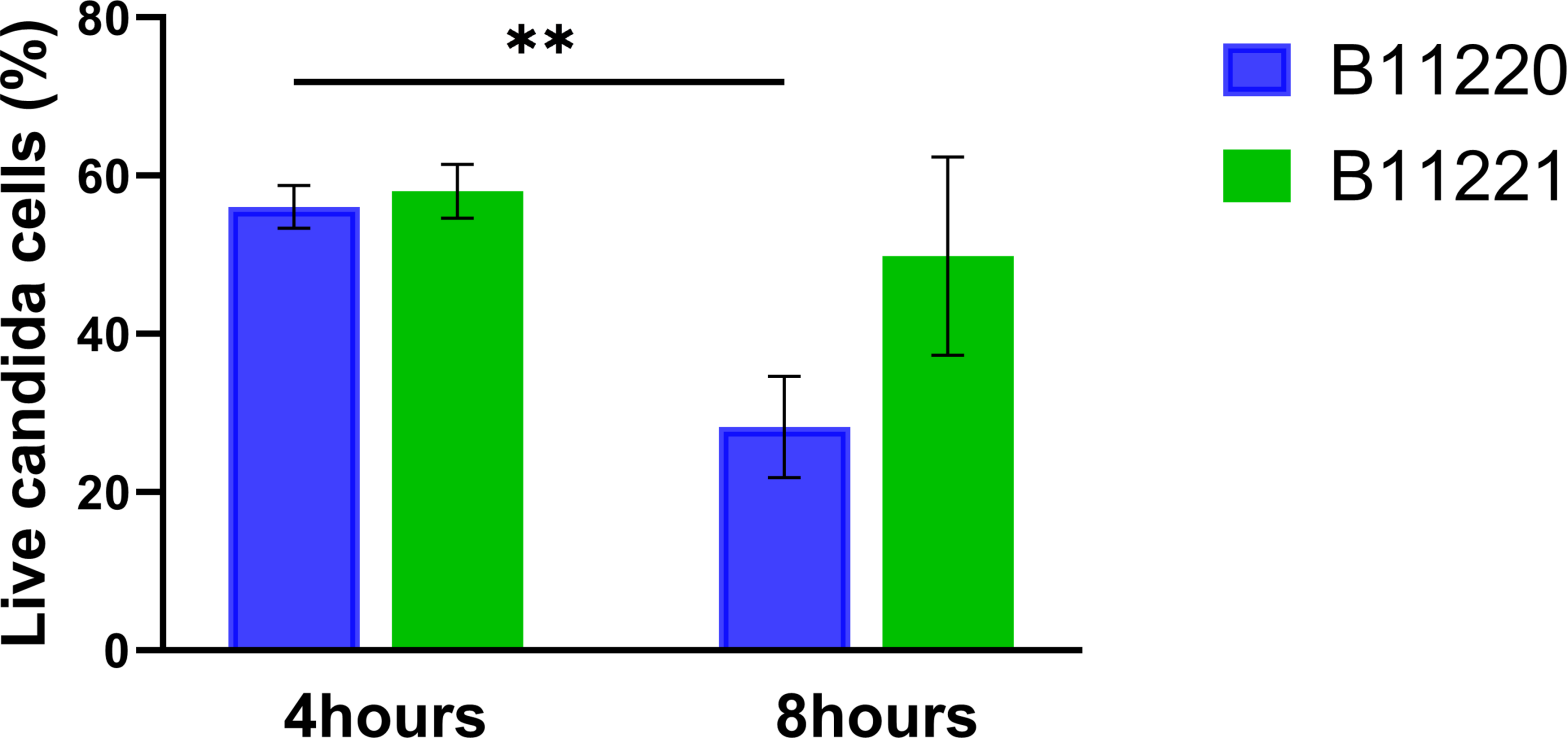
Survival of two *C. auris* isolates with macrophages. Candida survival quantification of two isolates incubated with macrophages for 4 hours and 8 hours, respectively. Bars are shown as mean ± SEM. Tukey’s multiple comparisons test (**P < 0.005).

Since we noted that cell wall genes were upregulated in D-galactose, and cell wall composition affects immune system evasion, we next investigated the interaction of the two *C. auris* isolate B11220^Δ^ and B11221 with macrophages. In mammals, macrophages are the first line of defense against microbial pathogens. We quantified candida survival after encounter with macrophage by measuring *C. auris* CFU over time. Survival of both isolates was reduced after 8 hours, but B11221 survival was significantly higher compared to B11220^Δ^ (Fig. 6). Gross microscopy revealed no obvious difference between the two isolates incubated with macrophages for 4 hours (Fig. S5). After 8 hours, more B11221 cells were unengulfed than B11220^Δ^, which is consistent with the survival. This suggests that B11221 evades macrophages better than B11220^Δ^.

**Fig 7:**
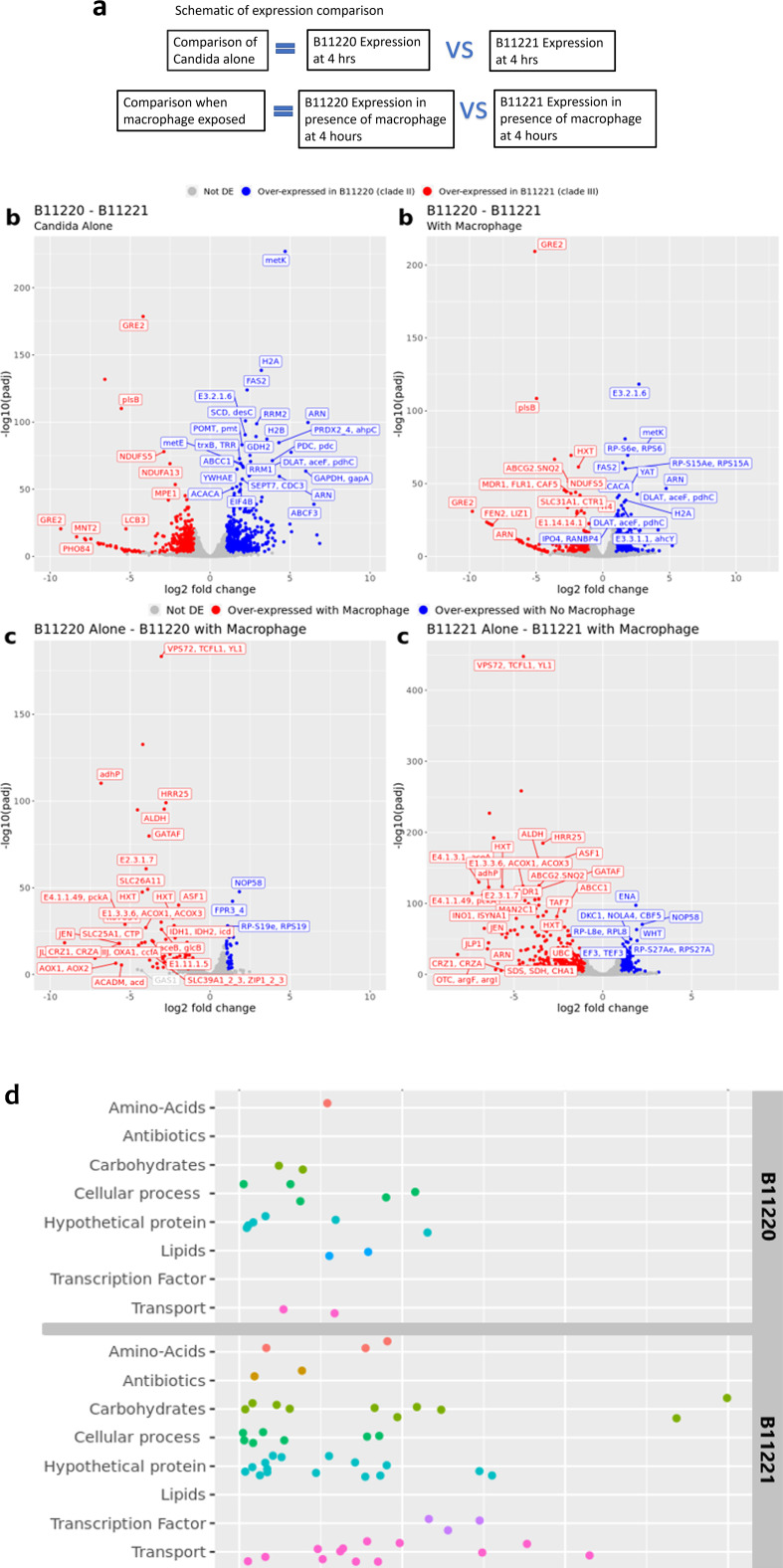
Transcriptomic analysis of the interaction between macrophage and the two *C. auris* isolates for 4 hours. (**a**) Schematic of expression comparison for RNA sequencing. For candida alone comparison, the expression of two isolates was compared at 4 hours culture without macrophage exposure; for comparison in the presence of macrophage, the expression of two isolates was compared after being exposed to macrophage for 4 hours. (**b**) RNA-seq volcano plots of log2 fold changes in B11220^Δ^ and in B11221 without (left) and in the presence (right) of macrophages (MΦ). Genes were differentially expressed (color coded) with a fold change > 1 and an FDR-adjusted *P* value < 0.05. Genes with a positive log2 fold change (blue) are over-expressed in B11220^Δ^ and under-expressed in B11221. Genes that have a negative log2 fold change (red) are over-expressed in B11221 and under-expressed in B11220^Δ^. (**c**) RNA-seq volcano plots of log2 fold changes in B11220^Δ^ (left) and in B11221 (right) exposed to MΦ. The genes were differentially expressed (color coded) with a fold change > 1 and an FDR-adjusted *P* value < 0.05. Genes with a positive log2 fold change (blue) are over-expressed without MΦ, genes that have a negative log2 fold change (red) are over-expressed in the presence of MΦ. (**d**) GO enrichment of genes was differentially expressed in the presence of macrophages but not differentially expressed without macrophages. Genes were differentially expressed with a fold change ≥ 2 and an FDR-adjusted *P* value < 0.05. Genes on the top part (positive log2 fold change) are upregulated in B11220^Δ^ and downregulated in B11221. Genes on the bottom part (negative log2 fold change) are upregulated in B11221 and downregulated in B11220^Δ^.

RNA sequencing was performed on 10 samples: two from each isolate pre-macrophage and three post-macrophage exposures. Volcano plots of DEGs of the two isolates revealed similar transcriptome patterns between the two isolates in conditions both without (561 vs 438) and with (259 vs 275) macrophage exposure (Fig. 7b; Fig. S6B). When exposed to macrophages for 4 hours, isolate B11220^Δ^ showed more upregulated genes than without macrophages (113 vs 35), and B11221 showed the same pattern (460 vs 263) (Fig. 7c). There were 357 unique genes significantly upregulated during macrophage exposure in isolate B11221 and only 11 unique genes in isolate B11220^Δ^. When comparing across isolates, B11221 had 124 unique upregulated genes, and only 82 in B11220^Δ^ (Fig. S6A). These data suggest that both *C. auris* isolates upregulate different genes in the encounters with macrophages and isolate B11221 activates more genes.

More genes were upregulated in isolate B11221 in transport and transcription factors at higher levels than isolate B11220^Δ^ (Fig. 7d), including *GAL4*-like transcription factors and sugar transporters, suggesting the transcription and transport activities were upregulated in B11221 during encounter with macrophages.

We further investigated whether genes already implicated in immune system evasion were upregulated (Table 2). Gliotoxin (GliC) is a known immunosuppressant mycotoxin that enables *A. fumigatus* to escape macrophages (85). Previous genomic approaches have identified an ORF (CJI97_001079) coding for a cytochrome P450 monooxygenase in the GliC-like family (86). We identified proteins similar to GliP, GliG, GliI, GliT, and GliN in both B11220^Δ^ and B11221 isolates but GliK and GliJ were not identified. These genes were not found to be significantly differentially expressed when exposed to macrophages. We also found two genes (CJI97_000658, CJI97_004624) that are identified as NADPH-dependent methylglyoxal reductase (EC 1.1.1.283), which is part of methylglyoxal degradation. These are significantly over-expressed in isolate B11221 compared to isolate B11220^Δ^ (5.10 and 9.79 log2 fold-change, with FDR-adjust *P*-value of 1.22e-91 and 3.51e14), but not significantly overexpressed in B11221 in the presence of macrophage. However, we found that an ORF encoding Candidapepsin (CJI97_001086), a virulence factor that degrades host proteins (87), was overexpressed in B11221 (4.15 log2 fold-change, FDR-adjusted *P*-value 0.0492).

**TABLE 2:** ORFs unique to *C. auris* B11221 and their upregulation in the presence of macrophages^*a*^

ORFs	Function	In the presence of macrophages	
		log2 FC	q-value
CJI97_001079	Cytochrome P450 monooxygenase	ns	
CJI97_001086	Candidapepsin	4.15	0.0492
CJI97_000658/CJI97_004624	NADPH-dependent methylglyoxal reductase	ns	
RTCAU38886/RTCAU37887	Exo-alpha sialidase/hyphal-regulated cell wall protein	3.59/6.67	7.08e-29/1.09e-24

^*a*^ FC: log2 fold change; q-value: FDR-adjusted *P*-value; ns: non-significant.

Finally, two of the most upregulated genes in isolate B11221 over B11220^Δ^ when both isolates are in the presence of the macrophage were found to be RTCAU38886 (8.56 FDR-adjusted *P*-value of 1.36e-10) and RTCAU37887 (log2 fold-change 8.71 with FDR-adjusted *P*-value of 5.78e-11). They were identified as exo-alpha sialidase (EC 3.2.1.18)/hyphally regulated cell wall protein with a domain similar to that of HYR1 in *C. albicans* that is shown to evade innate immune response induced during host interaction (85, 86). Both these genes are also significantly overexpressed when isolate B11221 is challenged in macrophage assay (3.59 and 6.67 log2 fold change, FDR-adjusted *P* values of 7.08e-29 and 1.09e-24). Taken together, these results show that isolate B11221 is more responsive to macrophage exposure, and that response activates candidapepsin and a HYR1-like gene that could mediate improved macrophage survival.

### *C. auris* isolate B11221 has lower β-glucan on the cell surface compared to isolate B11220^Δ^

Since several genes involving the cell wall were implicated in our carbon source study, we characterized the β-(1,3)-glucan exposure in the cell wall of *C. auris* B11220^Δ^ and B11221. β-glucan masking has been shown to affect fungal immune response (47, 88), and β-(1,3)-glucans make up approximately 40% of the *Candida* cell wall (46).

**Fig 8:**
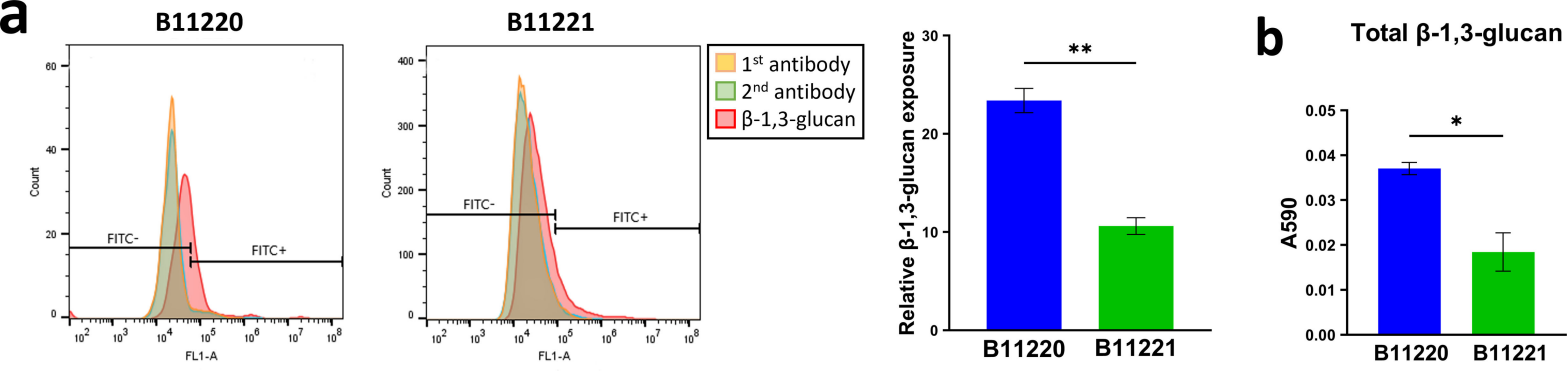
Quantitative analysis of cell wall β-1,3-glucan in two *C. auris* isolates. (**a**) Representative flow cytometry analysis of β-1,3-glucan exposure on *C. auris* cell wall. Yellow histograms correspond to control with only anti-β-glucan MAb, green histograms correspond to control with only anti-Mouse IgG secondary antibody, and the red histogram represents a sample with both antibodies. Red peak shifts on the plots from left to right indicate elevated fluorescence intensities indicating an increment in the β-1,3-glucan exposure. Quantitated results are shown on the right. (**b**) The total cell wall β-1,3-glucan content on B11220^Δ^ and B11221. Bars are shown as mean ± SEM. Unpaired *t*-test (*P < 0.05, **P < 0.005).

We compared the surface exposure of β-(1,3)-glucan between the two isolates using flow cytometry and found more β-(1,3)-glucans are exposed on the cell surface of B11220^Δ^ as compared to isolate B11221 (Fig. 8a). The differences in β-(1,3)-glucan exposure between the two isolates may contribute to their phenotypic variations. To eliminate the interference of other cell wall components masking the glucans, we also measured the total β-(1,3)-glucan present on the surface of the two isolates using ELISA. Our results indicate that isolate B11220^Δ^ contains more β-(1,3)-glucan (Fig. 8b), these results agree with our observation that isolate B11221 is more resistant to caspofungin than B11220^Δ^ (Fig. 3). The lower content of β-(1,3)-glucan required on isolate B11221 to maintain its normal function may enable it to be less affected by caspofungin which targets the biosynthesis of β-(1,3)-glucan. Furthermore, limited β-(1,3)-glucan exposure on isolate B11221 surface may lead to decreased recognition by macrophages thereby avoiding phagocytosis (Fig. S5) and better survival upon an encounter with macrophages as compared to isolate B11220^Δ^ (Fig. 6). Together, these results provide strong evidence that carbon metabolism and the cell wall are linked to antifungal resistance and immune system evasion in *C. auris* B11221.

## DISCUSSION

Since its emergence as a multi-drug resistant fungal pathogen in 2009, over 12 genomes of *Candida auris* have been reported (89). Here, we used a combination of short read (Illumina) and long read (Oxford Nanopore) sequencing techniques to assemble more complete genomes of two isolates of *C. auris* that represent the two ends of the drug susceptibility spectrum: the susceptible isolate B11220^Δ^ (Clade II) and the resistant isolate B11221 (Clade III). Using these improved assemblies, we confirm a large deletion that encodes a cluster of genes likely involved in the metabolism of alternate sugars. This cluster of genes is absent in Clades I, II, and IV, and present in Clades III and V (20). Clade III isolates are reported to be able to assimilate L-rhamnose, but not Clades I, II, and IV isolates (90), which may be because the gene cluster is missing in these clades. The Clade V isolate also conserved the gene cluster like Clade III isolates, supporting the hypothesis that in *C. auris* the absence of this cluster in Clades I, II, and IV is a loss rather than a gain in Clade III (20). High rate of genome rearrangements and sub-telomeric loss are also features reported unique to Clade II isolates (20), underlying their phenotype of being more sensitive to UV-C killing (91, 92) and susceptible to drugs. However, they are still potentially able to cause severe, drug-resistant infections as some isolates have been reported to acquire azole resistance (93). The fact that *C. auris* B11221 (Clade III) contains the gene cluster and can digest L-rhamnose is rare (94–96), and the loss of the L-rhamnose gene cluster in B11220^Δ^ may connect to its geological origins and difference in the hosts colonized.

In addition, our study identifies several unique ORFs in isolate B11221. These could contribute to the phenotypical differences between B11221 and B11220^Δ^. An ORF (CJI97_002126) for agglutinin (97), a multidrug resistance ABC transporter (CJI97_004171) (98), a hyphal-regulated cell wall virulence protein (RTCAU38886) (99), a sialidase (RTCAU37873) associated with macrophage survival and alternative sugar utilization (100), and formamidase (CJI97_002132) which is found in other human fungal pathogens but the role is not fully understood (96) to list a few examples.

We also looked for mutations in ORFs for glucan synthase, since they are responsible for resistance to echinocandin drugs (caspofungin) (32). We did not identify the resistance-associated hot spot region mutations in *FKS1* (101) in these two isolates. However, new FKS mutations related to drug resistance have been described (102) and a more sensitive and robust method has been developed (103), which can be applied in *C. auris* to detect either new mutations or non-*FKS1*-linked echinocandin resistance mechanisms.

Our transcriptomic studies also identify key rewiring and upregulation of genes in B11221 when grown on alternative sugars and when exposed to macrophages. We report a larger transcriptomic change in B11221 (Clade III) when metabolizing D-galactose as compared to B11220^Δ^ (Clade II). The activated genes are involved in galactose metabolism as well as cell wall composition and antifungal resistance. The D-galactose grown B11221 cultures lost aggregative form in a liquid medium, decreased adherence to the abiotic surface, became more drug susceptible, yet developed drug tolerance to caspofungin. The aggregation trait is linked to transcriptional changes in genes associated with surface adhesion in *C. auris* (104, 105), with potential clinical implications (81).

Previous studies on *C. albicans* reported that growth in galactose altered the outermost surface components of the fungal cell wall (106, 107), and consequently increased the synthesis of outer fibrillar-floccular layer mannoprotein adhesins (35, 108) that facilitate fungal adhesion and biofilm formation (108, 109); thus, the adhesion increases relative to growth in glucose (107, 110). By contrast, our results revealed decreased adhesion of B11221 in D-galactose and L-rhamnose, suggesting the metabolism of carbon source can be very different from *C. albicans* to *C. auris*, especially the isolates with the sugar metabolism gene cluster. Importantly, *C. auris* has an affinity for skin, unlike other *Candida* species that tend to colonize the gut. This increases the chances for *C. auris* to spread within and between healthcare networks when colonized or infected patients are transferred. Thus, decreasing attachment may contribute to its easy spread in healthcare settings (108, 109).

We also found that *C. auris* B11221 developed caspofungin tolerance when grown in the presence of glucose and galactose. The mechanisms of drug resistance and tolerance are different: Drug resistance directly affects the drug target or concentration in the cell so that the cell can grow in the presence of the drug; drug tolerance involves stress response pathways that indirectly affect cell growth, enabling cell survival despite the continued exposure to the drug. Drug tolerance is typically not genetic (not a mutation) since cells that are drug tolerant are often isogenic to those in the non-tolerant state (36). In the clinic, drug tolerance poses a significant barrier to the management of fungal infection because treatments can fail even when the isolate is susceptible to drugs *in vitro*, but can give rise to drug-tolerant microcolonies *in vivo*. Fungistatic drugs like fluconazole are often prescribed in combination with adjuvant drugs, to reduce tolerance without affecting resistance (36). Drug tolerance is thought to be a stepping stone to the evolution of drug resistance superbugs. Therefore, further study of drug tolerance and its mechanisms is important for the treatment of persistent candidemia. This study uncovers some new strategies that *C. auris* may use to become multidrug resistant. It can attach and survive longer on surfaces and utilize non-conventional sugars as it develops drug tolerance. Investigators are studying other *C. auris* variants that are associated with the drug tolerance phenotype (73).

The state of the host’s innate immunity plays a major role in the establishment of infections caused by opportunistic fungal pathogens such as *Candida* spp. Our results show that as early as 4 hours of interaction period, the two *C. auris* isolates survived macrophages at a similar percentage; however, phagocytosis observations at 4 hours revealed that B11220^Δ^ cells were already internalized by macrophages, as the bridges forming and stretching shapes were observed (Fig. S5, indicated by red arrow), while B11221 cells mostly attached around the surface of the macrophages and were not internalized. Our transcriptomic analysis shows that the interaction of macrophage cells with the two isolates is differentiated at an earlier stage. B11221 survived phagocytosis better than B11220^Δ^, and upregulated transcription factors and genes associated with transport when encountering macrophages as compared to B11220^Δ^.

There could be two basic strategies that enable *Candida* to survive macrophage encounters: (i) escaping from macrophage digestion so *Candida* can survive phagocytosis or (ii) reducing the recognition by macrophage receptors so they escape phagocytosis. We present evidence that isolate B11221 has less surface β-glucan and survives macrophages better supports the latter hypothesis. However, the former strategy cannot be completely ruled out. Even though it is not differentially expressed, the GliC-like family ORF (CJI97_001079) is expressed, making it possible that *C. auris* is synthesizing gliotoxin, an immunosuppressant mycotoxin (85). In either case, it is clear that *C. auris* B11221 rewires its transcriptome and expresses key genes involved in macrophage evasion, and these genes are influenced by growth on D-galactose.

To conclude, this study demonstrates that two *C. auris* isolates representing two distinct clades have evolved effective immune evasion strategies, environmental nutrient adaptation, and stress responses to ensure its host colonization, especially for *C. auris* B11221 which belongs to the Candidiasis outbreak-causing clade. This study also identifies genes involved in the virulence mechanisms of *C. auris*, a multidrug-resistant fungal pathogen, and provides genetic evidence linking metabolism, drug tolerance, and immune system evasion.

## Data Availability

The sequence data for *C. auris* B11220 and *C. auris* B11221 isolates are available on NCBI under BioProject PRJNA865346. *C. auris* B11220 has been assigned the NCBI BioSample accession SAMN30106616. The whole-genome assembly can be accessed with accession JANQCM000000000. The raw reads are available at the NCBI Sequence Read Archive (SRA)—nanopore reads are available under accession SRR20993210 and Illumina reads are available under accession SRR20993211. *C. auris* B11221 has been assigned the NCBI BioSample accession SAMN30106621. The whole-genome assembly can be accessed with accession JANQCN000000000. The raw reads are available at the NCBI Sequence Read Archive (SRA)—nanopore reads are available under accession SRR20993402 and Illumina reads are available under accession SRR20993403. RNA sequencing data for *C. auris* B11220 and *C. auris* B11221 are available at the NCBI. The accession number for the Sequence Read Archive (SRA) data is PRJNA902676.

## SUPPLEMENTAL MATERIAL

Supplemental figures iai.00103-24-s0001.pdf Figures S1 to S6.

ASM does not own the copyrights to Supplemental Material that may be linked to, or accessed through, an article. The authors have granted ASM a non-exclusive, world-wide license to publish the Supplemental Material files. Please contact the corresponding author directly for reuse.
