# Supplementary material for: A correlative study of the genomic underpinning of virulence traits and drug tolerance of Candida auris
Source: Infect Immun. 2024 May 9;92(6):e00103-24. doi: 10.1128/iai.00103-24 (PMC11326119; doi:10.1128/iai.00103-24)

Figure S1: Growth of two *C. auris* isolates B11220$^{\Delta}$ and B11221 on different sugar sources.

Serial diluted colonies growth of B11220$^{\Delta}$ (top rows) and B11221 (bottom rows) on SC agar in 8 sugars. OD600 of colonies from left to right are 0.1, 0.01,0.001, 0.0001.

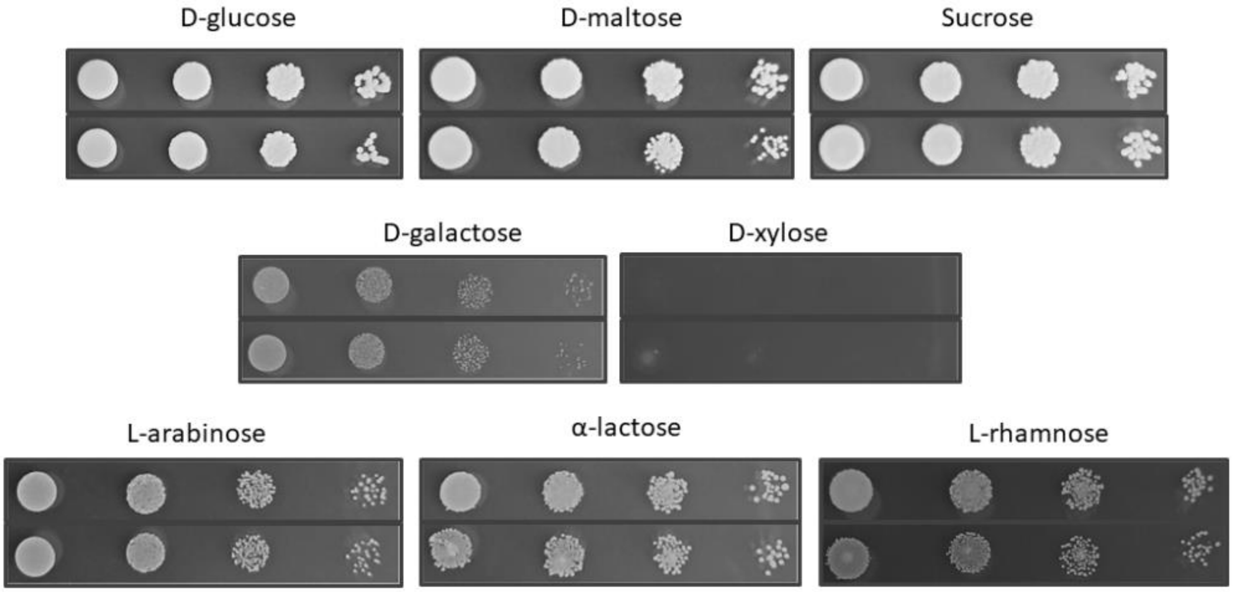

Figure S2: Adhesion assay characterization of B11220$^{\Delta}$ and B11221 on agar plates on various carbon sources.

Residue of two *C. auris* strains colony patches before and after washing off from SC agar with three sugar sources. The data shown reflects three independent replicates.

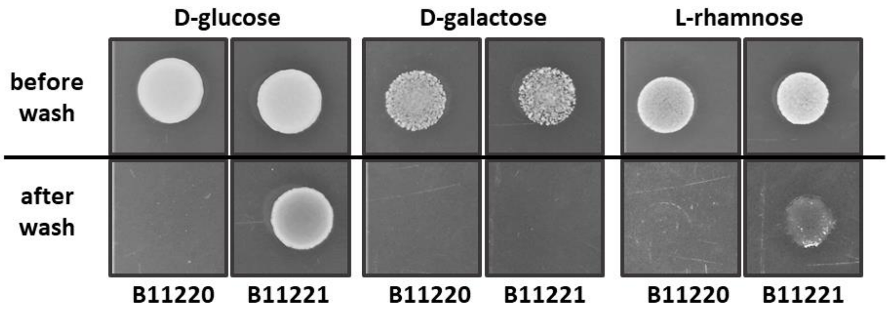

Figure S3A Significantly differentially expressed gene set differences within strains between carbon sources.

B11220 in Glu, Set of genes up regulated in B11220 in the presence of glucose compared to galactose; B11220 in Gal, Set of genes up regulated in B11220 in the presence of galactose compared to glucose; B11221 in Glu, Set of genes up regulated in B11221 in the presence of glucose compared to galactose; B11221 in Gal, Set of genes up regulated in B11221 in the presence of galactose compared to glucose.

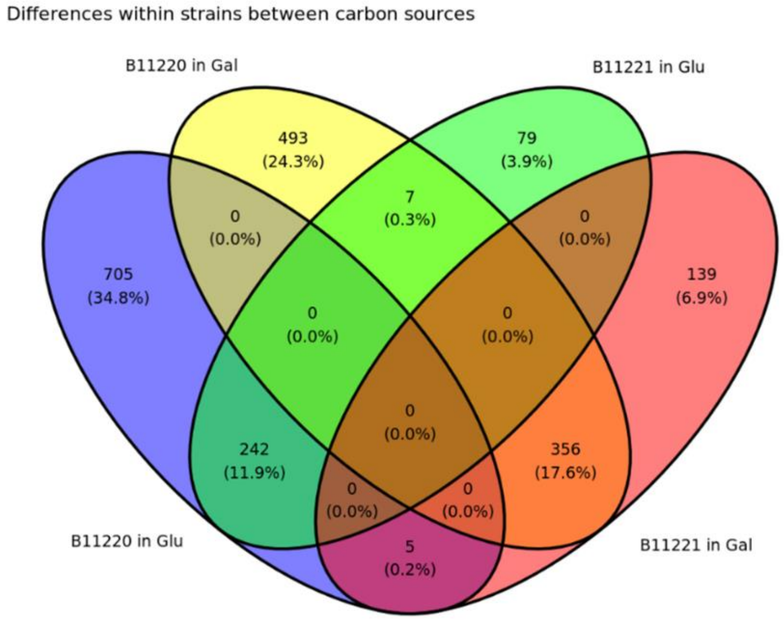

Figure S3B Significantly differentially expressed gene set differences between strains in different carbon sources.

B11220 over B11221 in Glu, Set of genes up regulated in B11220 compared to B11221 in the presence of glucose; B11220 over B11221 in Gal, Set of genes up regulated in B11220 compared to B11221 in the presence of galactose; B11221 over B11220 in Glu, Set of genes up regulated in B11221 compared to B11220 in the presence of glucose; B11221 over B11220 in Gal, Set of genes up regulated in B11221 compared to B11220 in the presence of galactose.

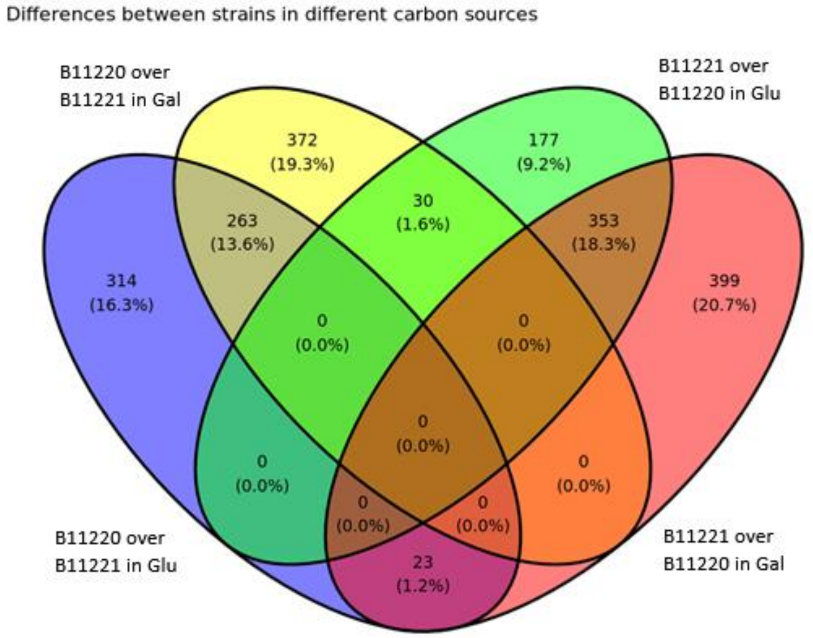

Figure S4A KEGG Pathway analysis of Ribosome. Darker shades of blue are down-regulated in isolate B11220$^{\Delta}$ grown in D-galactose compared to isolate B11221.

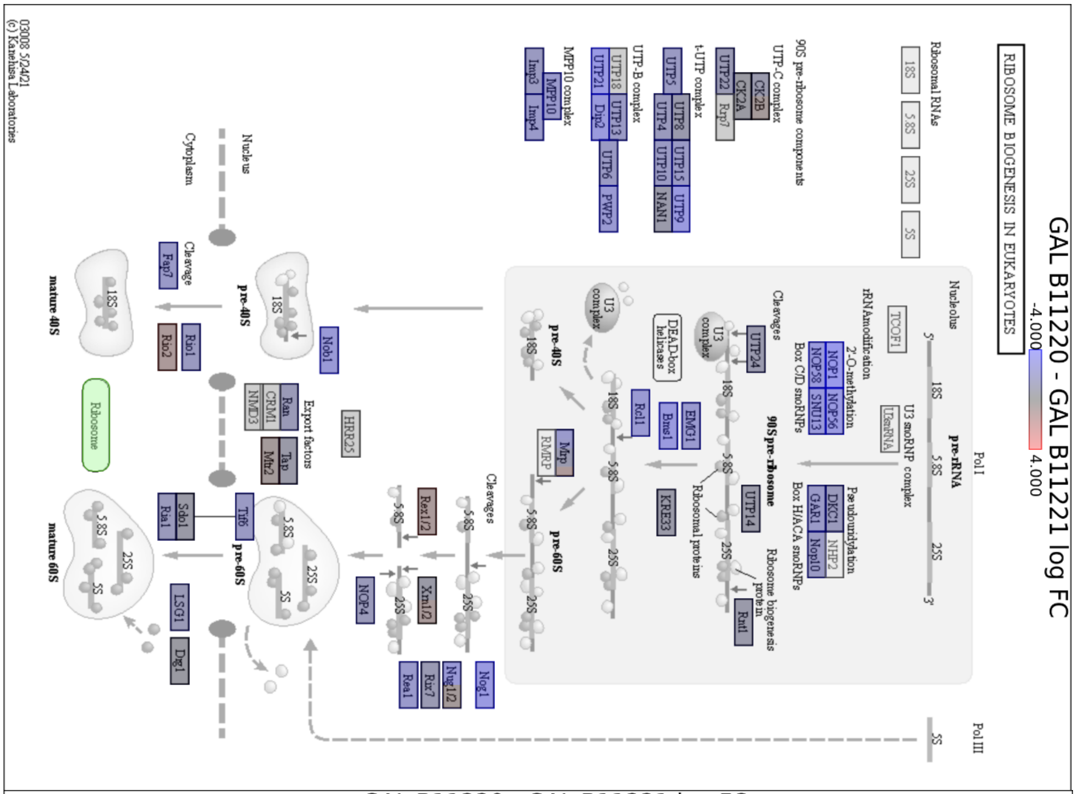

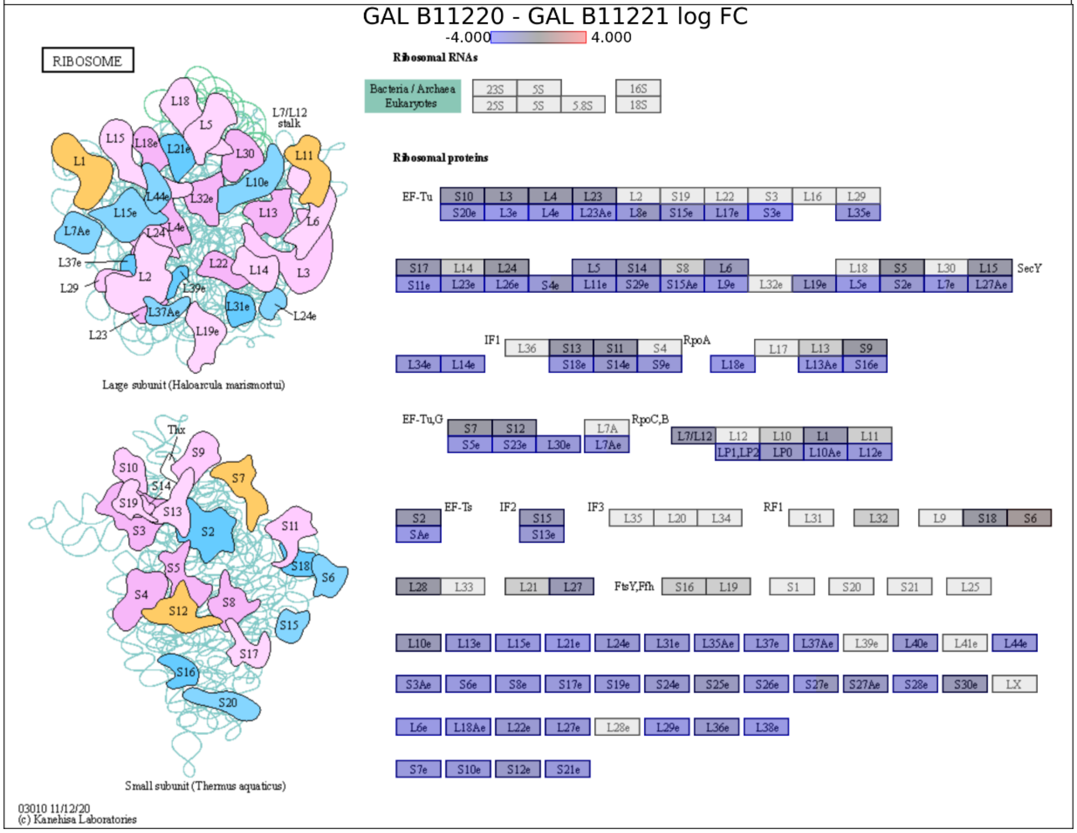

Figure S4B: Gene Ontology (GO) Term Enrichment for protein translation genes Function Namespace between two *C. auris* isolates B11220$^{\Delta}$ and B11221 grown on D-galactose.

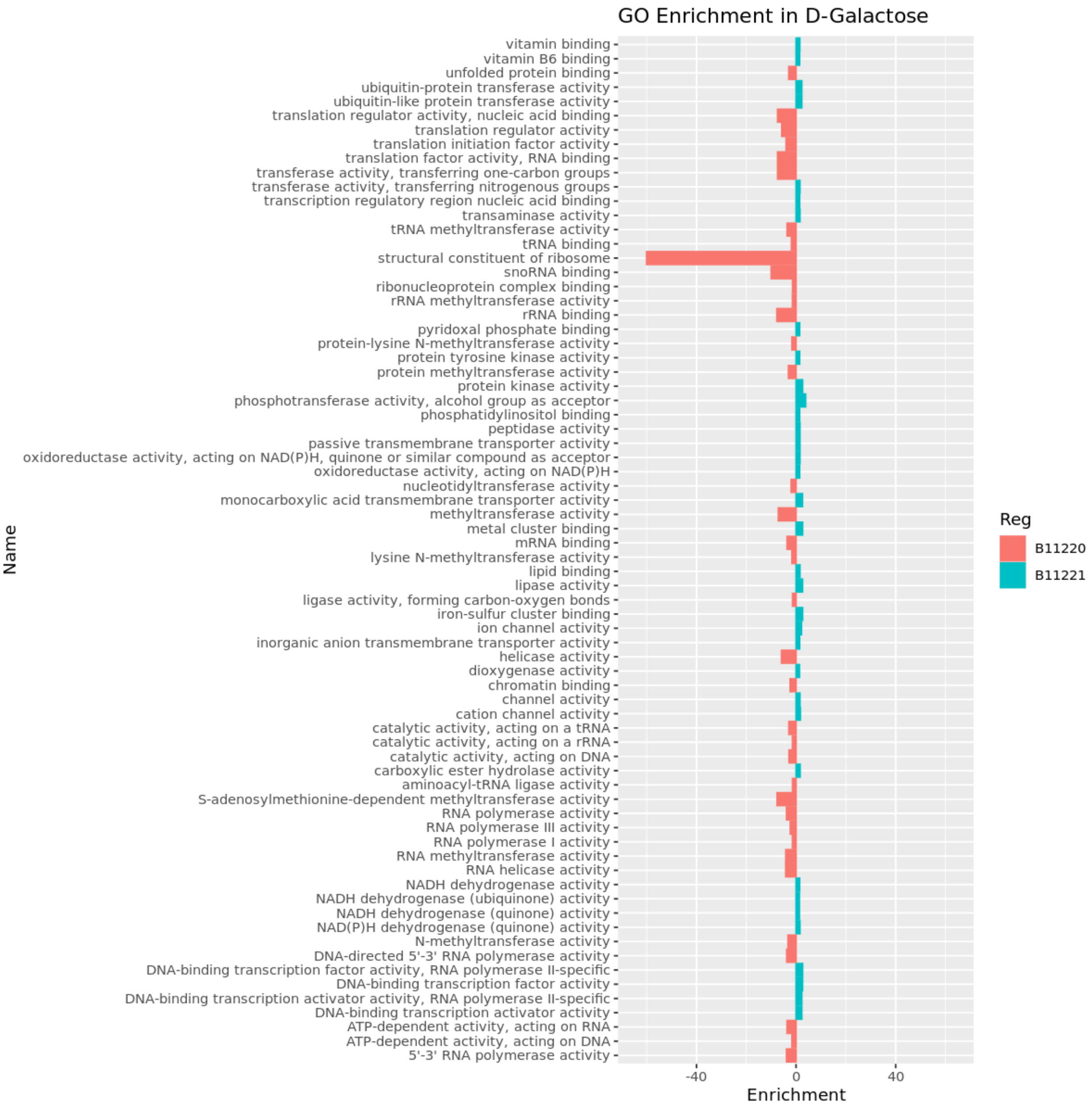

Figure S5: Growth of the two *C. auris* isolates B11220$^{\Delta}$ and B11221 in the presence of macrophages

(a) Microscopy of B11220$^{\Delta}$ (left panels) and B11221 (right panels) incubation with macrophages for 4 hours (top panels) and 8 hours (bottom panels). Non-internalized Candida cells are smaller oval shaped cells around macrophages, which are larger irregular shaped cells in photos. Magnification 400x. (b) Microscopy of B11220$^{\Delta}$ (left panels) and B11221 (right panels) incubation with macrophages for 4 hours (top panels) and 8 hours (bottom panels) after washing off floating cells. Red arrows indicate sites of *Candida* cells internalized, macrophage cells bridges formed and stretched. Green arrows indicate sites of *Candida* cells unengulfed. Magnification 600x. Scale bars represent 10 μM.

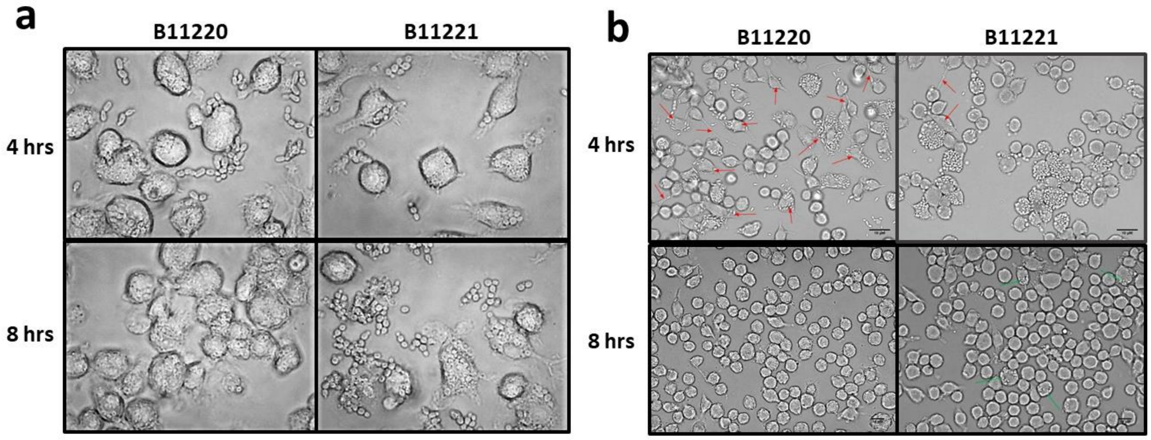

Figure S6A Significantly differentially expressed gene set differences across strains in the presence or absence of macrophage

B11220 over B11221 Absent, Set of genes up regulated in B11220 compared to B11221 while not in the presence of the macrophage; B11221 over B11220 Absent, Set of genes up regulated in B11221 compared to B11220 while not in the presence of the macrophage; B11220 over B11221 Mac, Set of genes up regulated in B11220 compared to B11221 while in the presence of the macrophage; B11221 over B11220 Mac, Set of genes up regulated in B11221 compared to B11220 while in the presence of the macrophage.

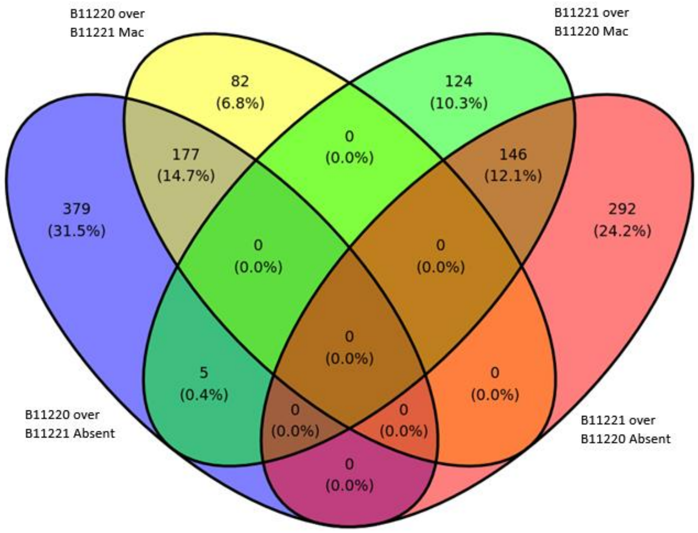

Figure S6B Significantly differentially expressed gene set differences within strains in the presence or absence of macrophage

B11220 Absent over Mac, Set of genes up regulated in B11220 absence of macrophage compared to present of macrophage; B11221 Absent over Mac, Set of genes up regulated in B11221 absence of macrophage compared to present of macrophage; B11220 Mac, Set of genes up regulated in B11220 in presence of macrophage compared to absence of macrophage; B11221 Mac, Set of genes up regulated in B11221 in presence of macrophage compared to absence of macrophage.

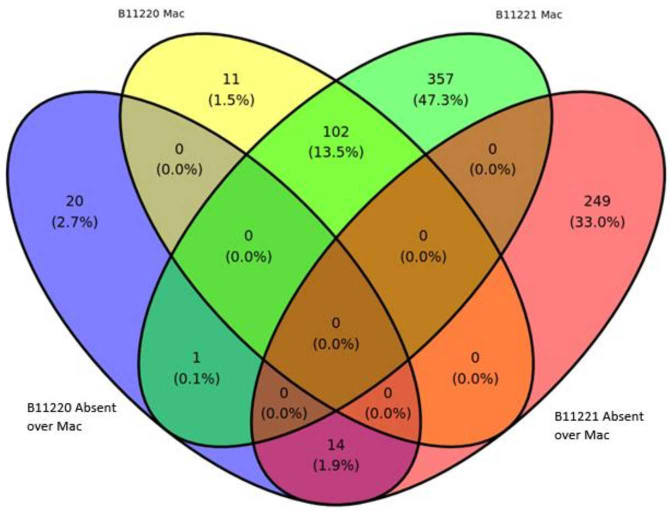

Supplement: Supplemental figures — Figures S1 to S6. [file iai.00103-24-s0001.pdf]
